# Global Analysis of the Human Pathophenotypic Similarity Gene Network Merges Disease Module Components

**DOI:** 10.1371/journal.pone.0056653

**Published:** 2013-02-21

**Authors:** Armando Reyes-Palomares, Rocío Rodríguez-López, Juan A. G. Ranea, Francisca Sánchez Jiménez, Miguel Angel Medina

**Affiliations:** 1 Department of Molecular Biology and Biochemistry, Faculty of Sciences, University of Málaga, Málaga, Spain; 2 CIBER de Enfermedades Raras (CIBERER), Málaga, Spain; University of California, Los Angeles, United States of America

## Abstract

The molecular complexity of genetic diseases requires novel approaches to break it down into coherent biological modules. For this purpose, many disease network models have been created and analyzed. We highlight two of them, “the human diseases networks” (HDN) and “the orphan disease networks” (ODN). However, in these models, each single node represents one disease or an ambiguous group of diseases. In these cases, the notion of diseases as unique entities reduces the usefulness of network-based methods. We hypothesize that using the clinical features (pathophenotypes) to define pathophenotypic connections between disease-causing genes improve our understanding of the molecular events originated by genetic disturbances. For this, we have built a pathophenotypic similarity gene network (PSGN) and compared it with the unipartite projections (based on gene-to-gene edges) similar to those used in previous network models (HDN and ODN). Unlike these disease network models, the PSGN uses semantic similarities. This pathophenotypic similarity has been calculated by comparing pathophenotypic annotations of genes (human abnormalities of HPO terms) in the “Human Phenotype Ontology”. The resulting network contains 1075 genes (nodes) and 26197 significant pathophenotypic similarities (edges). A global analysis of this network reveals: unnoticed pairs of genes showing significant pathophenotypic similarity, a biological meaningful re-arrangement of the pathological relationships between genes, correlations of biochemical interactions with higher similarity scores and functional biases in metabolic and essential genes toward the pathophenotypic specificity and the pleiotropy, respectively. Additionally, pathophenotypic similarities and metabolic interactions of genes associated with maple syrup urine disease (MSUD) have been used to merge into a coherent pathological module.

Our results indicate that pathophenotypes contribute to identify underlying co-dependencies among disease-causing genes that are useful to describe disease modularity.

## Introduction

**Figure 1:**
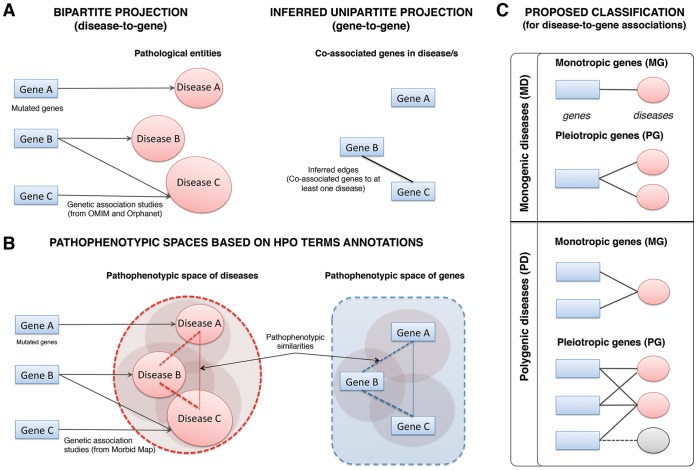
Schematic representation of distinct disease-to-gene relationships. Different disease associations between genes using (A) the data from genetic disease databases or (B) their associated pathological phenotypes. (A) The co-associations of genes in disease/s allow the inference of gene-to-gene projection (unipartite) from the disease-to-gene projection (bipartite). In this case Gene B and Gene C are co-associated with Disease C. (B) The HPO annotations of genetic diseases allow the description of pathophenotypic space for genes and calculation of the semantic similarity (pathophenotypic similarity) between them. In this case, novel relationships emerge as occur between Gene A and Gene B or Gene A and Gene C. (C) The proposed classification in this work: monogenic disease and monotropic genes (MD-MG), monogenic disease and pleiotropic genes (MD-PG), polygenic disease and monotropic gene (PD-MG), polygenic disease and pleiotropic gene (PD-PG). It is noteworthy that genes present in the MD-PG subset can also be present in the PD-PG subset (dashed line linked to monogenic disease in grey).

Phenotypes are the result of the expression of specific genetic backgrounds submitted to the influence of changing environmental conditions [1]. Thus, both the development and resulting symptoms of a given pathology are conditioned by interacting elements at multiple interconnected levels (from molecular to social levels) [2]. These complex interactions can be represented as networks to be analyzed using the principles of Network Theory [3]–[6]. In this sense, Network Medicine emerged as a new field to study the relationships among diseases and disease-causing genes [7]. Generally, data from genetic association studies establish the basic information for these analyses. Most of these data are available from different public repositories, for instance, Online Mendelian Inheritance in Man (OMIM) [8] and Orphanet [9]. This information can be projected onto networks also known as diseasomes (i.e. “the human disease network” and “the orphan disease networks”) [10], [11]. These diseasomes open the possibility to work on different types of network projections, treating networks as graphs, which can be used to detect emergent information. For instance, disease-to-gene associations represent bipartite edges (two different types of nodes in every edge) and conform a bipartite graph (as shown in the schematic representation in Figure 1A). On the other hand, projections of gene-to-gene edges and disease-to-disease edges can be inferred from the initial bipartite graph as two different “unipartite” graphs (each with only one type of node). Hence, edges in both inferred unipartite graphs represent either genes associated by a same disease (Figure 1A) or diseases associated through a same gene (these edges were not considered in this study), respectively. The first type of projections (gene-to-gene) are disease-causing gene networks and the second ones (disease-to-disease edges) are generally known as disease networks [10], [11]. Network-based methods enable us to find disease modules that may be understood as all molecular relationships involving disease-causing genes and other genes related to the same pathological processes [7]. In fact, several different biomolecular interactomes based on physical, metabolic or functional interactions have been used to capture some frames of the biological complexity associated with pathologies [12]–[17]. In this case, one of the most direct applications of network medicine approaches lies in the systematic exploration of the molecular mechanism shared by “apparently” distinct diseases [7]. The emergence of relationships among genes and diseases contribute to obtain more holistic views of the disease origin and environment, to predict new disease-causing genes [17], and possibly to locate new targets for disease diagnosis and/or intervention. All these challenges take part in a wider emergent discipline known as Systems Medicine [18].

However, current pathognomonic classifications are influenced by the traditional clinical procedures used during the 19^th^ century following Osleŕs principles [19]. These traditional procedures often tend to overvalue the most evident manifested abnormalities (pathophenotypes), causing a direct impact on how pathophenotypic profiles of patients are registered in the clinic [19]. Although it could help the diagnosis, many others pathophenotypes will go unnoticed. As a consequence, most genetic diseases are described as conceptual entities, pathologies, with certain specific clinical features. The disregard of pathophenotypes implies a considerable technical problem for network medicine based methods, since they can be primary consequences of the genetic disturbances. At present, to solve this problem standard phenotypic platforms are required to explore the underlying molecular and cellular mechanisms related to genetic predisposition in developing diseases [20]. Nevertheless, some previous works have claimed that the systematic phenotyping procedure requires ontologies to improve biomedical insights on functional gene communities [21]–[23]. In this case, the use of ontologies can be an interesting advance in the biomedical integration of this information. The Human Phenotype Ontology (HPO) represents a formalization of the semantic relationships [21], [24] among different clinical features described in OMIM (abbreviations used throughout the manuscript are reported in Table 1). Although HPO was initially developed to study the phenotypic associations in order to achieve a potential diagnostic use [25], this standardized biomedical knowledge on human abnormalities allows the identification of functional gene-to-gene relationships involved in similar pathological processes [26]. Recent studies conclude that the phenotypic similarity measurement proposed by Robinson and co-workers [25] has a significant contribution to the biological coherence compared to text-mining methods [27]. Therefore, on the one hand, the study of the similarity among pathologies requires representing them as a set of pathophenotypes instead of a pathological entity. On the other side, pathophenotypic information can be used to reinterpret the relationships among diseases identifying a new pathological phenotypic space that makes it possible the study of novel gene-to-gene associations (as can be seen in the schematic representation in Figure 1B). Zhang et al. [11] have recently stressed some limitations of network-based methods suggesting that the relationships between rare diseases cannot be fully captured by gene-to-gene projections alone. Therefore, the efforts to characterize the genetic and functional environment of given diseases (disease modules) can contribute to enrich the usefulness of disease network analyses.

**Table 1:** List of abbreviations used throughout the paper.

Abbreviation	Description
HPO	Human Phenotype Ontology
OMIM	Online Mendelian Inheritance in Man
HDN	Human Disease Network (bipartite projection)
ODN	Orphan Disease Network (bipartite projection)
HDGN	Human Disease Gene Network (unipartite projection)
ODGN	Orphan Disease Gene Network (unipartite projection)
MD-MG	Monogenic Disease and Monotropic Genes
MD-PG	Monogenic Disease and Pleiotropic Genes
PD-MG	Polygenic Disease and Monotropic Genes
PD-PG	Polygenic Disease and Pleiotropic Genes
PSGN	Pathophenotypic Similarity Gene Network
PIN	Physical Interaction Network
MGN	Metabolic Gene Network
FSGN	Functional Similarity Gene Network

In this work, network medicine approaches have been used to study the pathological relationships among genes using semantic similarities (that in this case are pathophenotypic similarities) instead of inferred unipartite edges (gene-to-gene) from bipartite edges (disease-to-gene associations). For instance, a classification of four distinct disease-to-gene associations is proposed (Figure 1C) to illustrate possible limitations of the current disease-to-gene network models [10], [11]. These classes provide four different subsets of genes in agreement with the number of genes associated with a disease (monogenic or polygenic) and the number of diseases associated with a gene (monotropic and pleiotropic). We have also built a pathophenotypic similarity gene network (PSGN) using semantic similarity [25] between genes that are annotated in HPO. The topological features of gene subsets obtained from inferred pathological networks have been analyzed and compared in PSGN. Additionally, the representation of PSGN in three different human biomolecular interactomes based on physical interactions, metabolic flux coupling and functional interactions were also evaluated. For this, a network comparison analysis [28] and a subsequent performance validation have been used to study the degree of contribution of each biomolecular interactome to the biological consistency of gene-to-gene pathophenotypic similarities. In addition, this biological coherence can be used to incorporate novel components in disease-causing gene modules, as we demonstrate for maple syrup urine disease (MSUD), an inborn error of the metabolism of branched-chain amino acids.

Summarizing, this work provides evidence that a standard phenotypic profiling expands the genetic disease associations using a specific ontology for human abnormalities. These pathologic relationships among genes were not obvious and, consequently, disregarded in previous disease network analyses.

## Methods

### Unipartite Projections of Current Diseasomes

#### Human disease causing gene network

In the present study, we worked on an updated version of the “Human Diseases Network” (HDN) [10] using Morbid Map from OMIM (http://www.omim.org/). HDN represents a bipartite projection of edges with two types of nodes, genes (MIM genes) and diseases (MIM phenotypes and genes/phenotypes) as described in OMIM. We followed a similar methodology to the one described by Goh et al. [10]. We retrieved all disease-to-gene associations where molecular bases are known and we discarded those phenotypes without MIM numbers. However, unlike previous works [10] we have not grouped diseases according to the similarity between their names. Here, each MIM phenotype or MIM gene/phenotype was considered as a pathological entity and each MIM gene was transformed to its respective Entrez Gene ID. This new version of the HDN consists of 2525 genes (Entrez Gene IDs) associated with 3132 OMIM entries (MIM numbers) generating a network of 5657 nodes and 3862 edges (HDN in Table S1). Hence, we built the respective unipartite projections based on inferred gene-to-gene relationships, named as human disease causing gene network (HDGN). This inference provides emergent gene-to-gene edges if genes are sharing at least one disease.

#### Orphan disease causing gene network

An updated version of the “Orphan Disease Networks” (ODN) [11] was built using Orphanet data. We used Orphanet because it is focused on genetic and low prevalent diseases; this database is actively updated and continuously reviewed by clinical experts. ODN is the bipartite projection of edges with two types of nodes, genes (Orpha numbers for genes) and orphan diseases (also in Orpha numbers for diseases). All those genes identified with Orpha numbers were transformed to Entrez Gene IDs. This new version of ODN consists of 2331 genes (Entrez Gene IDs) associated with 2125 genetic orphan diseases (ORPHA numbers) generating a network of 4456 nodes and 3657 edges (ODN in Table S2). In a similar procedure to that used for HDN (mentioned above), we built the unipartite projections based on gene-to-gene inferred relationships for ODN, named orphan disease-causing genes network (ODGN).

### Classification of Disease-to-gene Associations in Diseasomes

Both HDN and ODN were decomposed into four subclasses, based on the classification of the different types of disease-to-gene associations (Figure 1C): monogenic diseases associated with monotropic genes (MD-MG), monogenic diseases associated with pleiotropic genes (MD-PG), polygenic diseases associated with monotropic genes (PD-MG) and polygenic diseases associated with pleiotropic genes (PD-PG). In the context of the present study, we use the expression “monotropic genes” to refer to genes that have been previously related to only one disease and the expression “pleiotropic genes” to refer to genes that have been previously related to two or more diseases. Each subclass contains a subset of genes (Tables S3 and S4 Supplementary material).

### Pathophenotypic Similarity Gene Network (PSGN)

**Figure 2:**
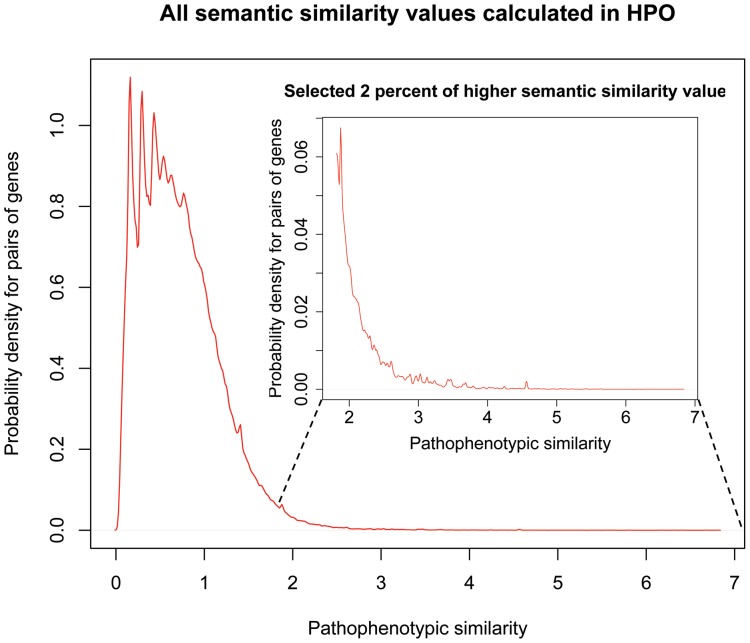
Probability density function for pathophenotypic similarities among pairs of genes in HPO. Densities of the pathophenotypic similarity values for all annotated genes in HPO (outer plot) and for the top 2% of gene pairs with the highest pathophenotypic similarities (inner plot). The bandwidth used was 0.01 and the pathophenotypic similarity value for the cut-off at the top 2% was 1.8179.

The pathophenotype gene network was built using pre-calculated values of semantic similarities between genes through the Human Phenotype Ontology (HPO). Previously, we had to describe the pathophenotypic space for genes as the set of clinical features (HPO terms) associated with each gene. Altogether 4669 diseases and 258 genes have direct annotations of their clinical features in HPO, so these diseases and genes have a list of HPO terms describing their phenotypic space. However, the lack of specific HPO terms regarding phenotypic abnormalities for many disease-causing genes hinders the explanation of their semantic relationships in the ontology. Many genes are annotated in the ontology with the sum of all HPO terms that describe their associated diseases in Morbid Map. In these cases, we used the file “gene_to_phenotype.txt” (available on HPO website) to link HPO terms and genes. This file was generated using Morbid Map associations between genes and diseases. Therefore, clinical features described in OMIM were translated in a standardized vocabulary of HPO terms (phenotypic abnormalities) that have been used to define a pathophenotypic space. As mentioned above, this pathophenotypic space for a gene can be directly annotated in HPO or indirectly annotated by the diseases associated with the gene in Morbid Map. We used the phenotypic space of genes to calculate their pathophenotypic semantic similarities with other genes. Only HPO terms with maximal information were used in agreement with the ontology properties and distribution of terms (see semantic similarity calculations section below). We discarded those branches of the ontology without an explicit description of phenotypic abnormalities such as “mode of inheritance” and “onset and clinical course”. We obtained a large pathophenotype gene network based on all semantic similarities between genes sharing HPO terms annotated in the phenotypic abnormality branch of the HPO. Despite an extensive literature review we could not detect a systematic methodology to calculate a cut-off score distinguishing between relevant or non-specific semantic similarities. Previous works used the semantic similarity to validate predictions or to evaluate shared biological features between highly specific subset of genes. However, in this case, we needed an optimal statistical threshold from which the signals, pathophenotypic similarities, should be out of the background noise. The cut-off will predetermine the topology of the network, so it could affect arguments and discussion about the “expansion” of pathophenotypic relationships respect to current unipartite projections (HDGN and ODGN). If we select a low similarity score we will introduce exponentially nonspecific relationships. In contrast, a very high score will constraint the model to already known pathological relationships. Therefore, we used the subset of known pathophenotypic similarities (gene pairs) in a binary classification system to estimate the optimal statistical threshold (see supplemental methods and discussion in Methods S1). Finally, the number of unspecific similarities was reduced by selecting the cut-off at the 98^th^ percentile that corresponds to the top 2% of significant gene pairs with higher semantic similarity values. To assess this clustering process of PSGN in the top 2% of phenotypic similarity, we plotted a kernel density distribution of probability of the pathophenotypic similarity for gene pairs (Figure 2).

### Biomolecular Interactomes

#### Physical interaction network (PIN)

We used the CRG Human Interactome as the reference for physical interaction network (PIN). This network of protein-to-protein physical interactions contains 10299 genes (Ensembl gene IDs) and 80922 interactions supported by evidence from at least one experiment [29]. The topological analysis of the largest connected component of the CRG Human Interactome was carried out under a similar procedure to that described in previous published works [30], [31]. However, all Ensembl gene IDs were transformed to Entrez Gene IDs to enable a node degree correlation and network comparison analysis with PSGN.

#### Metabolic gene network (MGN) based on metabolic flux correlations

Metabolic networks are usually based on different metabolic coupling approaches such as metabolite sharing (for instance, shared metabolites between enzymes) [15], [32], [33] and metabolic flux correlations (for instance, correlated metabolic enzymes by flux balance analysis) [34]. In this work, we used the flux-coupling metabolic network built by Veeramani et al. [34]. This network is based on the results of a flux balance analysis [34] of an updated version of the Human Metabolic network Recon 1 [35]. We built MGN using only these gene-to-gene interactions exceeding a metabolic flux correlation value of 0.1 and a “metscore” of 0 from the original network (Table S5, supplementary material).

#### Functional similarity gene network (FSGN) based on biological processes

The FSGN was built by using the measurement of the semantic similarity between genes described in the branch of biological processes of the Gene Ontology (GO). The functional space of a gene is represented by the set of GO annotations about the biological context where the gene is involved. Thanks to these annotations, genes are directly linked to biological processes describing all the functional features direct or indirectly related to genes. Classical semantic similarity measurements were used to calculate functional similarities between genes according to their functional space. In a similar procedure used for PSGN we removed unspecific functional associations in FSGN generated by irrelevant semantic relationships. However, there are great differences in the number of annotations between HPO and the branch of biological processes of GO. In this case, the main concern is that it resulted in huge size of this dataset. Therefore, we preferred to be quite more restrictive for this threshold, by taking as cut-off the 99.5^th^ percentile instead of the 98^th^. Thus we selected the top 0.5% of gene pairs with higher functional similarities (Figure S1).

### Semantic Similarity Score Calculations (Gene-to-gene)

The way to assign terms to objects is to add annotations. In the present case, the objects represent genes and terms corresponding to phenotypes (HPO terms) or biological processes (GO terms). The specificity of the terms associated with genes allows us to calculate the most significant relationships between them, which use to be related to its proximity to the root. The method we have chosen to calculate the semantic similarity between objects annotated is mainly based on the classical Resnik’s measurement [36]. This approach uses the information content (IC) concept that is a way to estimate the specificity of a term [25] and can be defined as the negative natural logarithm of the probability of a term
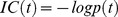
(1)where p(t) is defined on the basis of its frequency (number of term annotated) and the total of terms annotated in the ontology.



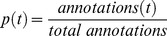
(2)If the probability decreases then the information content increases and consequently the specificity and the informativeness increase too. Thus, the IC tends to increase as we move away from the root to more specific terms.

For t1 and t2 terms in the ontology, the semantic similarity proposed by Resnik is defined as:
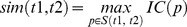
(3)where S(t1,t2) is the set of the shared parents of t1 and t2. In other words, the semantic similarity between two terms corresponds with the information content of the most informative common ancestor (MICA) [36].

#### Functional Semantic Similarity

Many studies so far have made a comparison between semantic similarity measurements using the Gene Ontology, but it seems that there is not a gold standard for semantic similarity measures between set of GO terms. In this work we use:
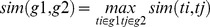
(4)a measurement that has been successfully used in some previously published works [37], [38]. In (4) g1 and g2 represent genes, where each one is related with a set of ontological terms. The semantic similarity value between sets of terms is calculated by comparing each pair of terms (3), one term of each set, and determined from the maximum value of all pair comparisons.

#### Pathophenotypic Semantic Similarity

Human Phenotype Ontology is still a novel tool and there are not many works related to the calculation of semantic similarity for this data structure. We have chosen the method proposed by the HPO creators for the comparisons between phenotypic profiles [25]. For g1 and g2 two genes; their semantic similarity is defined as:
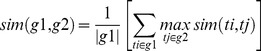
(5)where firstly is calculated the maximum value of IC, using the equation (3), between each term of g1 and the terms of g2. Finally, a set of values |g1| are used to work out their average.

The previous equation does not provide a symmetric matrix, since the calculated semantic similarity between g1 and g2 will not be the same as semantic similarity between g2 and g1, so Robinson and co-workers [25] suggest a symmetric version:
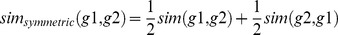
(6)


### Statistical Computing and Network Based Methods

All statistical computing, data management and graphics were performed in R, a free software environment. Network visualizations and their metadata analyses were performed in Cytoscape [39] and iGraph software, an R package (http://igraph.sourceforge.net/). Due to the large number of subsequent analysis of all built network, we provided a schematic workflow of all the essential steps followed for this study (Figure S1).

#### Network comparison analysis

Once all networks were built, we carried out a network analysis comparison to compute the nodal and edge intersection between PSGN and the rest of the built networks (HDGN, ODGN, PIN, MGN and FSGN). In the case of disease-causing gene networks (HDGN and ODGN unipartite projections of diseasomes), the intersection could provide a broad view of the similarity of these networks and the PSGN. Previously, we also calculated the intersection of edges between HDGN and ODGN to assess their mutual similarity. For biomolecular interactomes (PIN, MGN and FSGN) the nodal and edge intersection can be useful to explore the underlying molecular events of pathophenotypic similarities. However, biomolecular interactomes require two steps before the intersection analysis. First, we filter networks to ensure that both compared networks have only intersected nodes to minimize their differences in sizes (see schematic diagram of the process in Figure S1). All biomolecular interactomes were filtered to have genes with pathophenotypic data. Hence, we generated three biomolecular sub-networks that contain uniquely genes (nodes) participating in PSGN (Figure S1 and Table S6). This first step was essential for a more accurate value of the significance in the mutual coverage and to reduce the noise in the intersected edges. Moreover, this problem is bidirectional, so we used three different filters for PSGN (one for each cellular network). It will merge in three PSGN sub-networks (Figure S1 and Table S6). To evaluate the significance of the network comparison, we compared PSGN sub-networks with their respective randomized biomolecular interactome, treated and filtered exactly as the original networks. These randomizations were carried out preserving the node connectivity distribution in the respective cellular networks. Subsequently, we used NeAT [28] to compare networks treated as undirected ones. We used different metrics to identify the significance of the intersection: Maximal number of edges in the union, Jaccard coefficient and hypergeometric probability (p-value) [28], [40].

#### Network topological analysis

All gene (node) degrees were calculated for each pathological network and biomolecular interactome, using the iGraph software. Subsequently, a non-parametric test was used to study in each subset of genes the distributions of the node (gene) degree, the number of associated pathophenotypes per gene and the mean value of pathophenotypic similarity per gene. More precisely, a Mann-Whitney test was used to assess the significance of these distributions for gene subsets with the distributions of all genes in PSGN and their respective disease-causing gene network. This non-parametric test was run 1000 times for every subset of genes using a different random sample in each test. These random samples conserved the same size (number of genes) as their respective subset in the correspondent network. Subsequently, we calculated the mean p-value of all runs for every subset. Additionally, a Spearman's rank correlation test (α = 0.05) was used to analyze the degree of genes in HDGN, ODGN, PIN, MGN and FSGN with respect to the number of pathophenotypic relationships in PSGN.

#### Performance validation and ROC calculations

A binary classification system was used to analyze the performance of intersected interactions between different cellular networks (PIN, MGN, FSGN) and phenotypic interactions in PSGN. This binary classification is based on signal detection theory, using a receiver operating characteristic (ROC) analysis [41]. We compared biomolecular interactomes and their respective randomized versions (similar to those ones used in the network comparison analysis) with the PSGN using phenotypic similarities as the value of the signal (Figure S1). ROC curves were obtained considering the intersected interactions of PSGN with biomolecular interactomes as True Positives and those of PSGN with random biomolecular interactions as False Positives (Figure S1). We used randomizations to generate a dataset of False Positives proportional to the number of obtained True Positives for each biomolecular interactome. This procedure was useful to increase the confidence of the ROC analysis. In addition, we calculated the average area under the curve (AUC) for each interactome, calculating about 20 ROC curves following this same procedure.

## Results and Discussion

### Comprehensive Classification of Disease-to-gene Associations Contained in Currently Available Diseasomes

The projection in networks of the genetic associations data, available in OMIM and Orphanet, shows different patterns of connectivity among diseases and mutated genes (Figure 1A). Thus, we proceeded to build updated versions of existing models of disease networks, the “human disease network” (HDN) [10] and the “orphan disease network” (ODN) [11]. Subsequently, we classified all disease-gene associations of HDN and ODN in order to get an insight regarding their global distribution. For this purpose, we retrieved a total of 2525 and 2331 genes from HDN and ODN, respectively. Each gene dataset was subdivided in four different classes (Tables S3 and S4 for HDN and ODN respectively) according to our proposed criteria (Figure 1C): two monotropic classes (MD-MG and PD-MG) and two pleiotropic classes (MD-PG and PD-PG). Monotropic subsets are exclusive because their relationship with the disease is unique so genes take part in only one subset and they represent 72% and 69% of the total genes in HDN and ODN, respectively. In contrast, pleiotropic genes can be related to monogenic as well as to polygenic diseases so they can be present in both pleiotropic subsets.

The abundance of genes in each subset indicates how genetic association studies tend to distribute genes with different degrees of specificity for pathologies. In both networks, monotropic genes are found to be the most abundant ones, irrespective of the actual number of genes involved in the diseases (Table 2). For instance, “biunivocal” genes (MD-MG subset genes) represent over 56% and 30% of HDN and ODN genes respectively (Table 2). Even more, genes included in the PD-MG class are the most abundant ones in orphan disease network reaching 39% of the total genes. Many PD-MG associations could involve highly co-regulated genes (i.e. coding genes for different subunits of multi-protein complexes), so these genes can be considered a whole functional unit. In this case, we suspect that biunivocal relationships might be underestimated.

**Table 2:** Distribution of disease-to-gene associations on proposed classification.

	Human Diseases Network		Orphan Disease Networks	
Subset	Diseases per gene	Genes (%)	Diseases per gene	Genes (%)
MD-MG	1.00	1431 (56.7)	1.00	717 (30.8)
MD-PG	2.57	639 (25.3)	2.71	435 (18.7)
PD-MG	0.46	379 (15.0)	0.40	908 (39.0)
PD-PG^a^	2.13	371 (14.7)	1.68	584 (25.1)
All genes^b^	1.24	2525 (100)	0.91	2331 (100)

a Pleiotropic genes associated with at least one polygenic diseases.

b All genes in HDN and ODN respectively.

The ratios of diseases per gene agree with a pathological convergence (exclusive associations) and divergence (non exclusive associations) for monotropic and pleiotropic genes respectively (Table 2). These results are obvious taking into account our classification criteria. However, they provide a panoramic view of how a set of clinical features (pathophenotypes) observed in patients reach consensus and are attributed to a disease. These results seem to show a human annotation bias that can affect the current disease classifications.

### Features of Disease Causing Gene Networks (Unipartite Projections)

**Figure 3:**
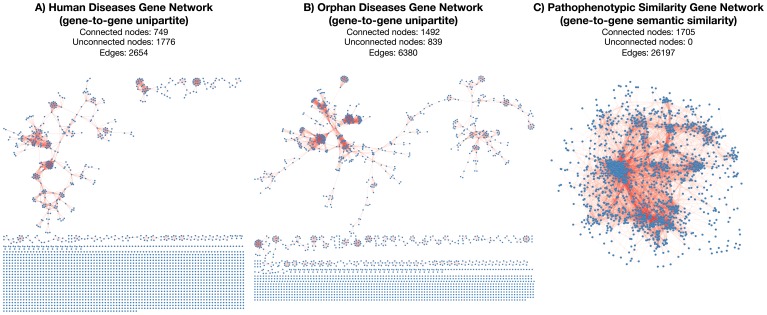
Unipartite gene-to-gene projections of the disease networks and the pathophenotypic similarity gene network. Human diseases genetic network (HDGN in panel A), Orphan diseases genetic network (ODGN in Panel B) and Human pathophenotype similarity gene network (PSGN in panel C). PSGN consists of one connected component (with a few unconnected genes), in contrast to HDGN and ODGN that show a great variety of isolated patterns of association. All unconnected genes (nodes) correspond to those uniquely associated with monogenic diseases, all of them were excluded in unipartite projections.

From the bipartite projections (disease-to-gene) of HDN and ODN, we built their corresponding unipartite projections (gene-to-gene) (as can be seen in Figure 1A), named as “human disease causing gene network” (HDGN) and “orphan disease causing gene network” (ODGN) respectively (Figures 3A and 3B). Both unipartite projections are based on the emergence of gene-to-gene relationships (edges) inferred from pair of genes co-associated with at least one disease (Figure 1A). Accordingly, all genes in the MD-MG subsets and those uniquely associated with monogenic diseases in MD-PG will appear as unconnected genes in unipartite projections (HDGN and ODGN).

HDGN include 749 genes (nodes) and 2654 inferred gene-gene relationships (edges) among them (Figure 3A and Table S1). However, ODGN is twice as larger as HDGN with 1492 genes and 6380 inferred gene-gene relationships (Figure 3B and Table S2). At first glance, the topological structures of unipartite networks (HDGN and ODGN) are quite similar (Figures 3A and 3B) although an enrichment of unconnected nodes in HDGN is clear when compared to ODGN (1776 and 839 for HDGN and ODGN respectively). This enrichment is mainly due to the higher number of biunivocal relationships (MD-MG) in HDGN (Table 2). Therefore, this is the reason why HDGN shows fewer inferred relationships (2654) than ODGN (6380).

We carried out an analysis of the intersection between both unipartite networks (HDGN and ODGN) to assess an estimation of their similarity. But first we removed all unconnected nodes because they were not considered structural components of these networks. The resulting intersection was 481 genes (intersected nodes) and 662 inferred gene-gene relationships (intersected edges) corresponding to 24% and 10% of edges in HDGN and ODGN respectively (Table 3). Both networks show a Jaccard coefficient of similarity (number of edges in the intersection divided by the number of edges in the union) of 7.9% (Table 3). Surprisingly, the similarity is lower than expected *a priori* which indicates strong differences between the two data sources (OMIM and Orphanet).

**Table 3:** Network intersection analysis between HDGN and ODGN.

Network features	Values
Number of nodes in HDGN	749
Number of nodes in ODGN	1492
Number of edges in HDGN	2654
Number of edges in ODGN	6380
Observed nodes in the intersection	481
Observed edges in the intersection	662
Percentage of edges in HDGN	24.94
Percentage of edges in ODGN	10.38
Jaccard coefficient of similarity	0.079^a^

a Fraction of edges in the intersection respect to the total edges in the union.

These results reinforce the hypothesis that the absence of a systematic procedure in the phenotypically characterization of genetic diseases will affect the utility of network medicine methods. In particular, it leads to the isolation of genes and diseases from their real pathological processes, making it practically impossible to identify groups or subgroups of related pathologies. This observed tendency to the exclusiveness (that is to say, the abundance of monotropic gene-disease relationships) considerably increases the disease-gene association specificity that may be of interest for genetic testing.

### Features of Pathophenotypic Similarity Gene Network (PSGN)

The exclusiveness mentioned above could affect pathological processes with many disease variants. In the case of these diseases, some genes play a primary role in the progression of the pathology but others modulate the phenotypic variability.

To tackle this problem, HPO offers possibilities for a formal study of the pathophenotypic relationships among genes on the bases of their semantic similarities (pathophenotypic similarities). Therefore, we defined the pathophenotypic space of each gene, consisting of the set of HPO terms associated with the gene (as shown in Figure 1B). These spaces were described using only specific HPO terms, those farthest terms from the root of the ontology, to calculate the semantic similarity value between every two given genes (see methods, Table S7). Higher values of semantic similarity indicate greater specificity in the common pathophenotypic space between a pair of genes. It is known that ontology-based phenotypic similarity methods can also contribute to improve disease-causing gene networks based on phenotypic information built with text-mining analysis [42] or random-walk trajectories between genes considering the ontology as a simple graph [43].

From all calculated pathophenotypic similarities greater than zero, we selected the top 2% of more significant pairs of genes. This selection provides the pathophenotypic similarity gene network (PSGN) with 1075 genes and 26197 gene-to-gene pathophenotypic similarities (Figure 3C and Table S7). Disease-causing gene networks (HDGN and ODGN) exhibit explicit structural differences when they are compared to PSGN (Figure 3); for instance, PSGN consists of only one giant connected component (Figure 3C), which is not the case for HDGN and ODGN.

Almost all the pathophenotypic gene annotations used in HPO originally come from OMIM and they represent the sum of all clinical features of diseases associated with a gene. Accordingly, the pathophenotypic similarity for a gene is somehow dependent on the number of diseases associated with this gene (see methods section). Hence, we proceed with a comprehensive study to assess whether the pathophenotypic similarity can be used to reinterpret the pathological relationships between genes (see supplementary methods and discussion in Methods S1).

### Pathophenotypic Similarity Reveals a New Understanding of Pathological Relationships

The survey of the mutual coverage between PSGN and each unipartite projection (HDGN and ODGN) was carried out with an analysis of their intersections.

The resulting intersections of PSGN with each unipartite projection proved 528 shared nodes and 1055 shared edges for HDGN and 931 and 1669 for ODGN (Table 4). Therefore, 39% and 26% of inferred pathological relationships intersect with pathophenotypic similarities of PSGN, even improving the intersection between disease causing gene networks (mentioned above). The Jaccard coefficient of similarity of the intersection of PSGN with each pathological network was 3.8% and 5.4% for HDGN and ODGN respectively (Table 4). This can be considered an interesting performance value if we take into account the dependence on the Jaccard coefficient on the different sizes of compared networks (the number of edges in the union are 27796 for HDGN and 30908 for ODGN). Furthermore, there are about 25000 new pathophenotypic similarities, excluding inferred pathological relationships, to be used for the discovery of new underlying pathological relationships among genes.

**Table 4:** Network intersection analysis between PSGN and HDGN or ODGN.

Network features	HDGN values	ODGN values
Number of nodes in PSGN	1705	1705
Number of nodes in pahtological network	749	1492
Number of edges in PSGN	26197	26197
Number of edges in pahtological network	2654	6380
Observed nodes in the intersection	528	931
Observed edges in the intersection	1055	1669
Percentage of edges in PSGN	4.03	6.37
Percentage of edges in pahtological network	39.75	26.16
Jaccard coefficient of similarity	0.038^a^	0.054^a^

a Fraction of edges in the intersection respect to the total edges in the union.

#### Topological analysis exhibits the emergence of unnoticed pathological relationships

**Figure 4:**
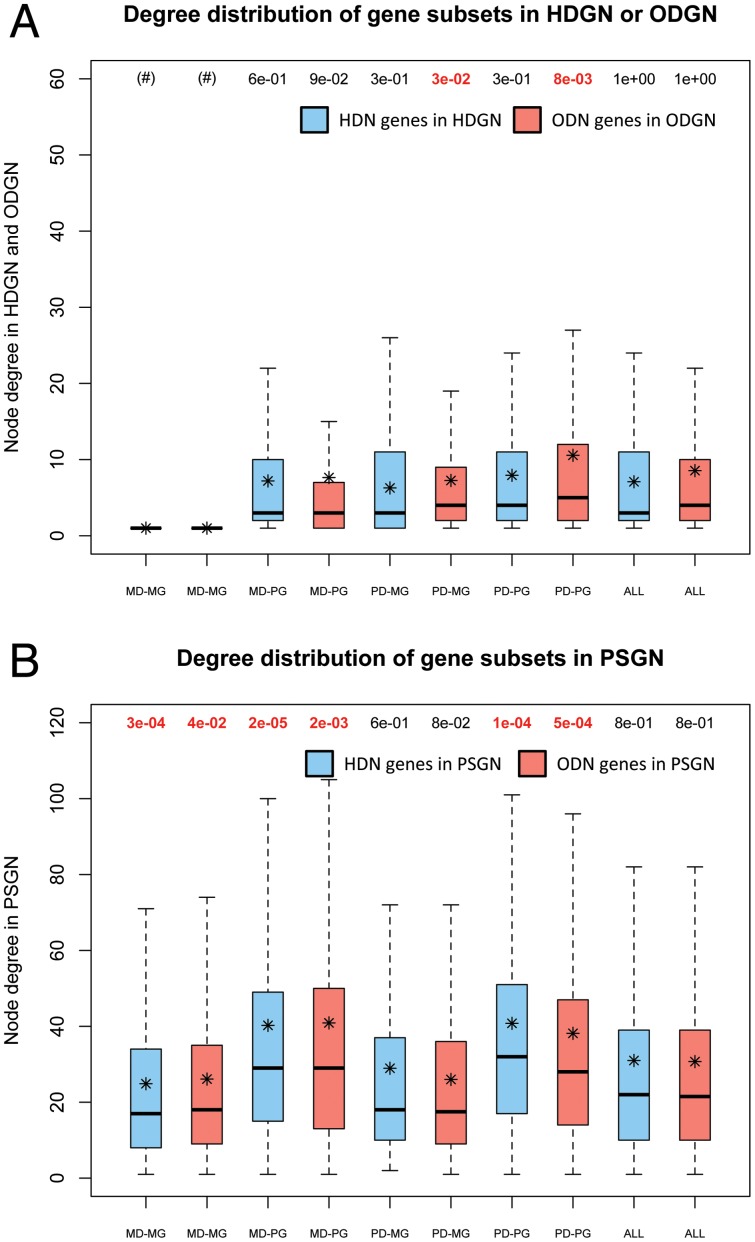
Degree distribution of subset genes in pathological and pathophenotypic gene-to-gene networks. Box plots of the degree of subset genes in HDGN (blue) and ODGN (red). Box plots of the degree of subset genes in PSGN for ODN subsets (blue) and for ODN subsets (red). In bold and red, significant p-values. (*) Mean values. (#) Subsets of completely unconnected genes.

We have also studied how genes in PSGN are distributed in comparison to HDGN and ODGN. Subsequently, we analyzed the degree distribution of genes for each network (HDGN, ODGN and PSGN), as well as for their respective gene subsets (MD-MG, MD-PG, PD-MG and PD-PG of HDN and ODN). We carried out a Mann-Whitney test to assess the significance of the difference of the degree distribution of each subset in their respective disease-causing gene network and in PSGN (Figure 4, a boxplot was used in all the cases). In agreement with our classification criteria, MD-MG genes (bi-univocal) have null connectivity in their respective disease-causing gene networks (Figure 4A). By contrast, MD-MG genes are phenotypically linked to a mean of 25 genes in PSGN indicating an expansion of pathophenotypic relationships between disease-causing genes in PSGN (Figure 4B). In pathological networks, degree distributions are significantly different for ODGN subsets (PD-MG and PD-PG) but not for HDGN subsets (see their correspondent p-values in Figure 4A). On the other hand, degree distributions in PSGN are quite similar when compared to the equivalent subsets of HDGN and ODGN, where higher node degree for pleiotropic genes and lower for monotropic genes can be appreciated (Figure 4B). In addition, Spearman's rank correlation test was used to explore degree correlations between the pathophenotypic similarity (PSGN) and disease-causing gene networks (HDGN and ODGN) (Table S8). Weak (but statistically significant) positive correlations were found between gene pathological and pathophenotypical relationships (Table S8). These results, as shown in Figure 4 and Table S8, clearly show that gene degrees in pathological networks differ from those calculated using pathophenotypic similarities.

The (apparently) most striking observation is that genes uniquely associated with monogenic diseases (genes in MD-MG and many of MD-PG) are present in PSGN. The vast majority of these genes appeared as unconnected genes in the unipartite projections of HDN and ODN (as shown in Figure 3). This means that pathophenotypic similarities lead to the emergence of novel relationships that remained hidden in the gene-to-gene projections of current diseasomes.

#### Specific contribution of gene subsets to gene-to-gene pathophenotypic similarities

In light of the result discussed above, we consider it necessary to prove the contribution of each type of gene subset to the gene-to-gene similarities of PSGN. This could help to unveil the relationship between the pathological convergences or divergences and the pathophenotypic similarities [30]. Therefore, we analyzed the abundance of pathological phenotypes and the average pathophenotypic similarity per gene.

**Figure 5:**
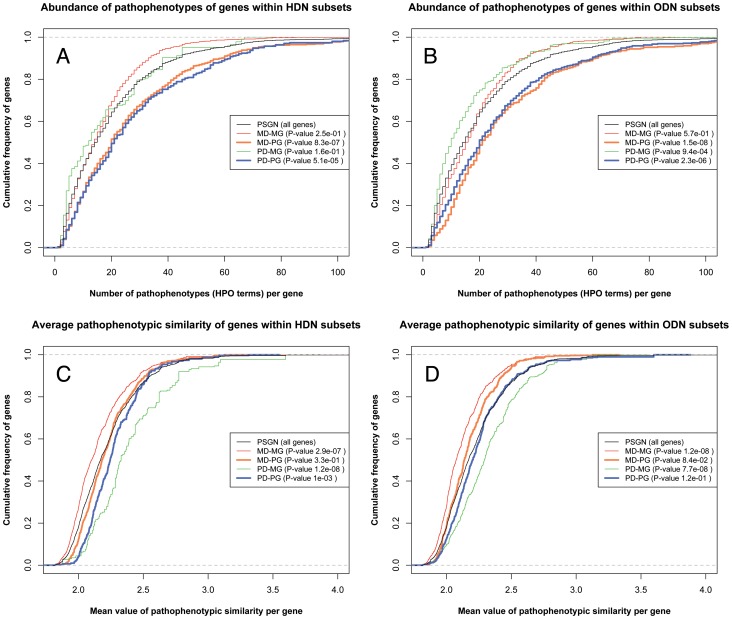
Distributions of the number of pathophenotypes and pathophenotypic similarities in each subset. MD-MG (red line), MD-PG (orange line), PD-MG (green line), PD-PG (blue line) and PSGN (Black line). Upper panels represent the cumulative frequency of the number of specific pathophenotypes annotated for genes in HDN (C) and ODN (D) subsets, the whole set of genes in HPO (PSGN) was used as the reference distribution. Lower panels represent the cumulative frequency of the average pathophenotypic similarity associated with genes in HDN (C) and ODN (D) subsets, the whole set of genes in HPO (PSGN) was used as the reference distribution. The p-values, included in each legend, represent the mean of the resulting p-values after 1000 non-parametric tests (Mann-Whitney test) where each subset was compared, each time, with a random sample of the pathophenome of the same size of the subset (see methods).

Figure 5 (panels A and B) represents the distribution of the abundance of pathophenotypes (HPO terms) in genes for HDN and ODN subsets. Pleiotropic genes show distributions significantly different to the distribution of all genes included in PSGN using a Mann-Whitney test (see their correspondent low p-values for MD-PG and PD-PG, Figure 5 panels A and B). On the other hand, monotropic genes seem to be well represented in the pathophenome (whole genes of PSGN) showing only slight differences in the distribution of PD-MG subset for ODN (see the p-value for PD-MG in Figure 5B). Consequently, we can be confident that the phenotypic descriptions used for monotropic genes are not underestimated and they are enough to calculate their pathophenotypic similarities to other genes. By contrast, as expected, pleiotropic genes tend to be annotated in the ontology with more clinical features compared to the whole gene annotations. For an overall estimation of how each subset contributes to the pathophenotypic co-dependence between genes, we calculated the average of pathophenotypic similarity values associated with each gene in the PSGN in order to compare their distributions in different subsets (Figures 5C and 5D). The monotropic subsets contain genes with the highest specific relationships to diseases. Nevertheless, monotropic subsets show very different behavior compared to all genes of the PSGN in the distribution of the average pathophenotypic similarities related to genes within HDN and ODN subsets (see the low p-values for MD-MG and PD-MG in Figures 5C and 5D). MD-MG subsets show lower average pathophenotypic similarity values (Figures 5C and 5D). As a result, these distributions also reveal pathophenotypic relationships among genes that remained lost in the gene-to-gene unipartite projections of HDN and ODN. The distributions of PD-MG subsets show higher average phenotypic similarities between genes (observe that the green curves in Figures 5C and 5D are displaced to the right when compared to the respective red curves, as well as to the rest of curves). This observation could be mainly due to the fact that they are sharing similar sets of annotations, and in many cases they are functional units or strongly co-regulated molecular complexes. With regards to pleiotropic subsets, they seem to be slightly affected by the number of genes involved in the disease (monogenic and polygenic). Nonetheless, their abundance of pathophenotypes could increase the number of non-specific relationships between genes. In this case, non-specific relationships will tend to show low values of similarities decreasing the average value associated with genes. In fact, this agrees with the higher connectivity observed for pleiotropic subsets in both HDN and ODN (Figure 4). For this reason, we analyzed the degree of association between the abundance of pathophenotype per gene and the average similarity value per gene. A weak Spearman correlation was obtained (p-value 1.8E−26 and *r_s_* = −0.25, Figure S2) so we can ensure no clear dependence between both parameters. However, there is a tendency to decrease the mean value of pathophenotypic similarity for genes with abundant HPO terms annotations.

Apparently, the use of semantic similarity measurements produces a rearrangement in the pathophenotypic co-dependence between genes overcoming the bias that can be introduced from the original source of data, the Morbid Map. However, the gene pleiotropy dampens their average pathophenotypic similarity values indicating a rise of unspecific relationships with other genes compared to monotropic genes. This observation reinforces our suggestion that the representation of diseasomes as unipartite projections is insufficient to study other underlying (and not necessarily obvious) pathophenotypic relationships.

### Overview of the Relationship between Metabolic or Essential Genes and Pathophenotypic Similarity

**Figure 6:**
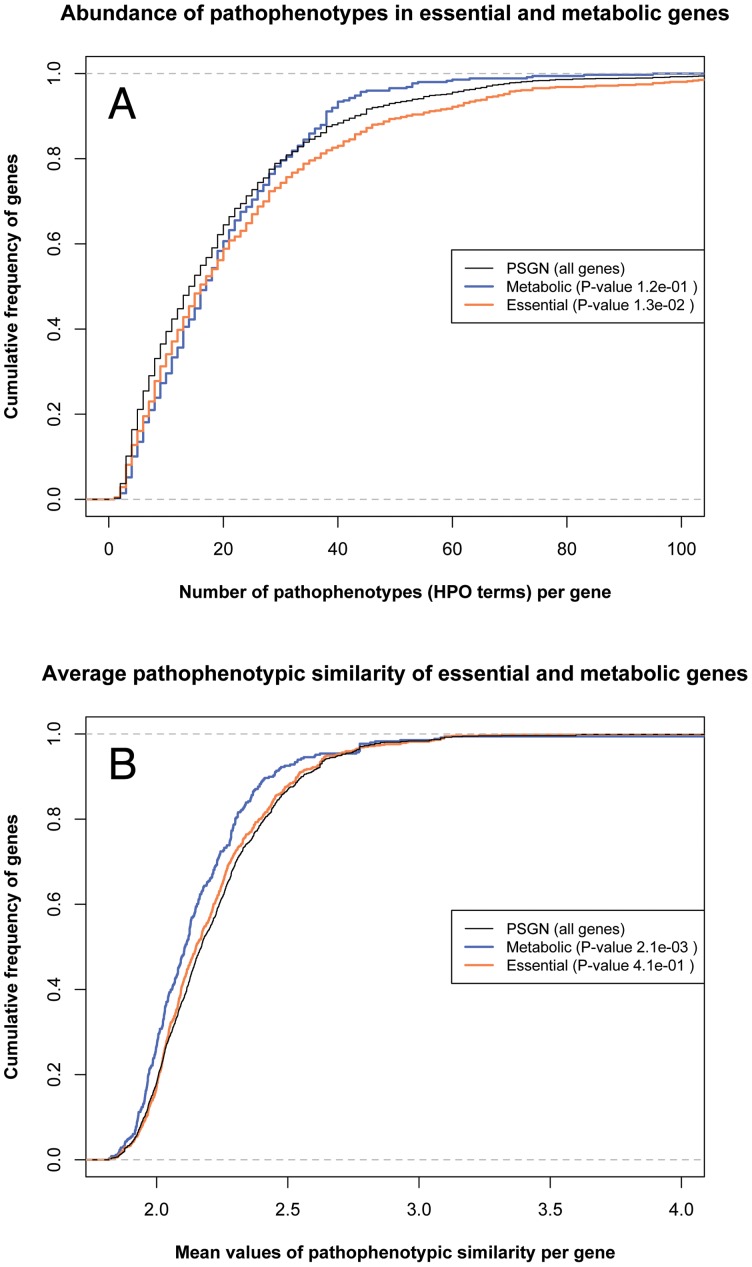
Distributions of the number of pathophenotypes and the pathophenotypic similarities for metabolic and essential genes. Metabolic genes (orange line), essential genes (orange line) and the PSGN (Black line). Upper panel (A) represents the cumulative frequency of the number of specific pathophenotypes annotated for genes, the whole set of genes in HPO (PSGN) was used as the reference distribution. Lower panel (B) represents the cumulative frequency of the average pathophenotypic similarity associated with genes, the whole set of genes in HPO (PSGN) was used as the reference distribution. The p-values, included in each legend, represent the mean of the resulting p-values after 1000 non-parametric tests (Mann-Whitney test) where every set of metabolic and essential genes was compared, each time, with a random sample of genes in PSGN of the same size of their respective set (see methods).

Taking into account that metabolic and essential disease genes represent about 18% and 34% respectively of the total disease-causing genes, we also studied how they are represented in each subset of genes in our classification (Table 5). The subsequent study of cumulative frequencies per gene of the associated pathopenotypes (Figure 6A) and the average pathophenotypic similarity values (Figure 6B) suggest that gene subsets tend to be associated with different biological properties.

**Table 5:** Distribution of essential and metabolic genes in current diseases network.

	HDN		ODN	
	Essential	Metabolic	Essential	Metabolic
Subset	genes (% in class)	genes (% in class)	genes (% in class)	genes (% in class)
MD-MG	409 (28.6)	308 (21.5)	219 (30.5)	202 (28.2)
MD-PG	315 (49.2)	79 (12.4)	228 (52.4)	64 (14.7)
PD-MG	106 (28.0)	65 (17.2)	245 (27.0)	105 (11.6)
PD-PG^a^	189 (50.9)	34 (9.2)	286 (49.0)	73 (12.5)
All genes^b^	856 (33.9^c^)	458 (18.1)	802 (34.4^c^)	409 (17.6)

We determined for each class the percentage of genes considered as essentials and metabolic coding genes included in the built metabolic network (MGN).

a Pleiotropic genes associated with at least one polygenic diseases.

b All genes in HDN and ODN respectively.

c Minimal changes are seen compared to Zhang et al.(2011) [11], these differences are due to updating of data Orphanet.

#### Enrichment of metabolic genes in the MD-MG subclass

Biunivocal classes (MD-MG) are markedly enriched in metabolic coding genes with respect to the other classes; on the contrary, PD-MG is underrepresented by metabolic enzymes. On the other hand, the pathophenotypes corresponding to metabolic genes do not differ from those of the whole pathophenome (see non-significant p value in Figure 6A). However, the mean value of phenotypic similarity is lower for metabolic genes than for the whole pathophenome (Figure 6B). For instance, metabolic genes tend to be involved in more specific pathological processes and exclusively related to pathophenotypes recognized as genetic diseases. It seems relevant that metabolic genes are mainly enriched in the MD-MG subset: 67% and 49% of the whole set of genes in MGN are MD-MG for HDGN and ODGN, respectively. In addition, metabolic genes show a lower distribution of the mean values of pathophenotypes compared to the whole pathophenome (Figure 6). Therefore, dysfunctions in metabolic genes prove a functional bias in disease and gene association studies toward the pathophenotypic specificity (Figure 6). At least two factors could contribute to explain this observation: first, the molecular basis of metabolic dysfunctions can be more precisely identified in these diseases; second, these diseases exhibit pathophenotypes with highly distinguishable features. In any case, both factors can be influenced by the application of routine biochemical analysis in the clinical setup, which allows an easier detection of abnormal concentrations of metabolites in blood or urine.

#### Enrichment of essential genes in the pleiotropic subsets

Zhang et al. [11] have reported an enrichment of essential genes in ODN with respect to HDN but our results suggest that both networks show a similar proportion of essential genes (Table 5). In particular, the results shown in Table 5 also indicate that an enrichment of essential genes is produced in pleiotropic gene subclasses. The number of pathophenotypes associated with essential genes is significantly higher than that obtained when using all genes in the PSGN (Figure 6A). But their distribution of mean values of phenotypic similarities is statistically indistinguishable from that of the whole pathophenome (Figure 6B). Some previous network medicine works have discussed how essential genes are represented in different diseaseomes [10], [11], [30]. Barabasi and co-workers concluded that disease-causing genes are not essential genes because their associated lethality could have severe consequences [10]. Chavali et al. [30] proposed two different topological features for phenotypically divergent genes and essential disease genes, inter-modular and intra-modular hubs respectively. Zhang et al. [11] in their analysis of the orphan disease network found that ODs are enriched in essential genes as compared with the whole set of diseases. In contrast, when we compared the same essential gene dataset used by these authors in the updated versions of HDN and ODN, no detectable differences were found (Table 5). Our observation differs from that of Zhang et al. [11], maybe due to the use an updated version of both disease-causing gene networks and the same dataset of essential genes. In any case, our results do not support the idea that there could be a negative correlation between gene essentiality and disease prevalence. Nonetheless, it seems that there is a certain enrichment of essential genes in the subsets of “pleoiotropic” genes, that is, those associated with more than one disease (Table 5). This result agrees with observed by Chavali et al. in the dataset of shared genes by diseases [30]. The dataset of essential genes used in these works [10], [11], [30] are human orthologous of lethal mouse genes catalogued in the Mouse Genome database [44].

From our point of view, the enrichment of essential genes in pleiotropic disease-causing genes leads to interesting evolutionary questions on how mutations in these genes are related to their lethality for other mammals and might be involved in the limits of human evolvability [45], [46].

### Integrative Analysis of PSGN

#### Built biomolecular interactomes (PIN, MGN and FSGN)

The heterogeneity of the cellular interactions among genes affects (either directly or indirectly) the progression of the diseases [13]. Thus, the disturbances caused by genetic mutations can be transmitted in biological systems in several distinct ways. Three different biomolecular interactomes were built to study the association between the pathophenotypic similarity and each type of biological interaction (physical, metabolic and functional interactions). PIN results in 9580 genes connected through 74657 physical interactions (Table S5). MGN contains 535 enzyme-coding genes interconnected by 9812 flux correlations (Table S5). The top 0.5% of functional similarities in the branch of biological processes in the Gene Ontology corresponds to FSGN. FSGN results in 9157 genes and 496973 significant functional similarities (Table S5). For each biomolecular interactome, we evaluated their coverage in PSGN and the contribution of each type of biological interaction to the score of pathophenotypic similarity.

#### Network comparison analysis between biomolecular interactomes and PSGN

‘A network intersection analysis was carried out using the PSGN as reference and the biomolecular interactomes (PIN, MGN or FSGN) as queries. Nevertheless, the observed differences in size and density of the studied networks could be the cause that the direct network comparison analysis would provide no useful significance values. Therefore, we decided to standardize the contents of the networks by using the intersection of nodes (see methods section) to minimize differences between the reference (PSGN) and the rest of the networks (PIN, MGN or FSGN). This step (Figure S1) provoked a strong structural decomposition from all the original networks that resulted in sub-networks (Table S6). Although we reduced the size differences between the intersected networks, other features are still preserved like the density of edges, which are inherent to the nature of each network (Table 6).

**Table 6:** Counts of nodes and edges in the comparison of PSGN and biomolecular interactomes.

		PIN		MGN		FSGN	
Symbol	Description	Nodes	Edges	Nodes	Edges	Nodes	Edges
R	Reference (PSGN)	1233	15550	131	321	1381	17233
Q	Query (biomolecular interactome)	903	1779	154	1060	1376	30318
QvR	Union	1240	16907	158	1257	1387	45078
ôR	Intersection	896	422	127	124	1370	2473
Q!R	Query not reference	7	1357	27	936	6	27845
R!Q	Reference not query	337	15128	4	197	11	14760

All calculations were performed using NeAT [28]. The query is PSGN and used reference corresponds to each biomolecular interactomes.

The network comparison results show statistically significant intersections of edges for all biomolecular interactome sub-networks compared to their respective PSGN sub-network (Table 7). This was not the case for randomized networks used as negative controls. The hypergeometric test shows a lower significance of the pathophenotypic similarities resulting in the intersection between PSGN and MGN when compared to PIN and FSGN (Table 7). Nevertheless, the Jaccard coefficient of similarity between biomolecular interactomes and their respective PSGN sub-network was higher for MGN and FSGN (9.8% and 5.4% respectively) than for PIN (2.5%). In this sense, both the percentage of edges remaining in the reference sub-network and the Jaccard coefficient of similarity seem to be good indicators of the size of the phenotypic space covered by the intersection (Table 7). The 23.7% of physical interactions between diseases-causing genes match with pathophenotypic similarities, 11.7% and 8.1% for metabolic flux correlation and functional interactions respectively. FSGN showed the largest and most significant coverage in PSGN (Table 7), which means that the functional relationships of genes based on biological processes define the broadest context of the molecular mechanisms associated with disease-causing genes. Concerning biochemical interactomes (PIN and MGN), PIN exhibits a greater coverage of genes at the intersection than MGN, although the latter presents the highest Jaccard coefficient of similarity (Table 7).

**Table 7:** Significance of the number of edges at the resulting intersection in the network analysis comparison.

			PIN		MGN		FSGN	
Symbol	Description	Formula	Network	Random	Network	Random	Network	Random
N	Nodes in the union	–	1240	1238	158	158	1387	1387
M	Max number of edges in the union	M = N*(N−1)/2	768180	765703	12403	12403	961191	961191
E(ôR)	Expected edges in the intersection	E(ôR) = Q*R/M	36.01	27.96	27.43	24.33	543.57	196.95
ôR	Observed edges in the intersection	–	422	35	124	17	2473	194
Q (%)	Percentage of query edges	perc_Q = 100*ôR/Q	23.72	2.54	11.70	1.81	8.16	1.77
R (%)	Percentage of reference edges	perc_R = 100*ôR/R	2.71	0.23	38.63	5.30	14.35	1.13
Jac_sim	Jaccard coefficient of similarity	Jac_sim = ôR/(QvR)	0.0250	0.0021	0.0986	0.0137	0.0549	0.0069
P value	P-value of the intersection	Pval = P(X> = ôR)	**4.0E−308**	1.1E−01	**2.7E−51**	9.6E−01	**1E−321** a	5.9E-01

All calculations were performed using NeAT [28]. The query is PSGN and used reference corresponds to each biomolecular interactomes. In bold, those significant p-values.

a The limit of precision for the hypergeometric test.

#### Specific contribution of biomolecular interactions to pathophenotypic similarities

Most of the published network biology studies have made use of the degree of a node (number of connections with other nodes) to assess its relevance in a network. In fact, node degree has been extensively used in physical interaction networks [10], [11], [30], [31] but also in metabolic networks [15], [32]. In this work, a topological analysis was carried out in different biomolecular interactomes to calculate the degree of genes (based on gene-to-gene interactions).

**Figure 7:**
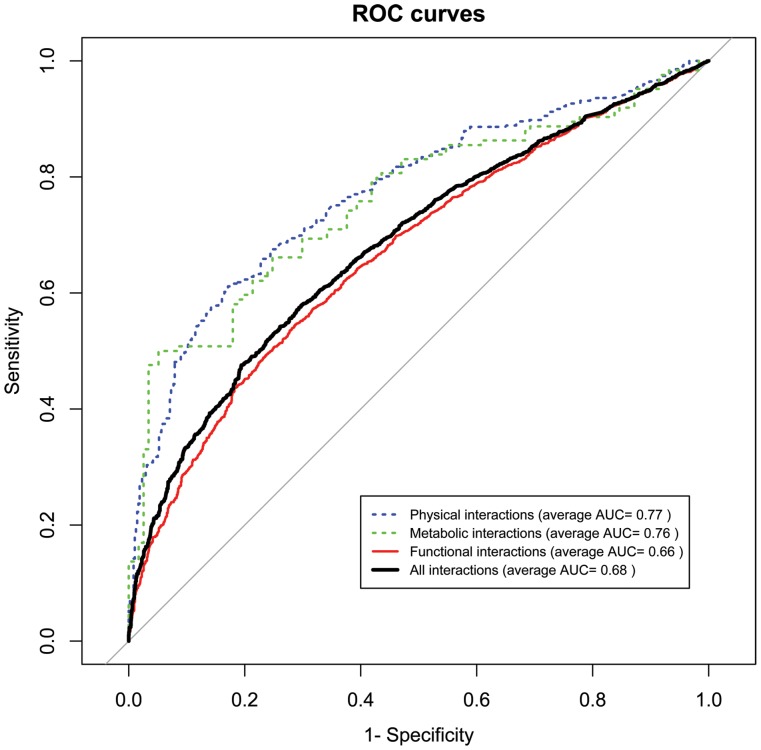
Receiver operative characteristic (ROC) curve performance by biomolecular interactions of pathophenotypic similarities. Physical interactions (dashed blue line), metabolic flux correlations (dashed green line), functional interactions (red continuous line) and an integrated interactome generated by the sum of all other interactomes (black continuous line). ROC curves were computed to assess the signal of pathophenotypic similarities for biological interactions. True positives (TP) were those interactions that where found in the intersection between PSGN and each biomolecular interactome (PIN, MGN and FSGN). The dataset of false positives (FP) was calculated from intersected gene pairs between PSGN and randomizations of each biomolecular interactome. We obtained severals different FP datasets to calculate the average area under the curve (AUC), it was 0.77 for PIN, 0.76 for MGN, 0.66 for FSGN and 0.68 for the integrated interactome. Only biochemical interactomes show significantly different AUCs to that of the integrated interactome (average p-values of 2.2E−6 and 4.1E−2 for PIN and MGN respectively).

To estimate whether the abundance of biological interactions for genes is correlated with the number of phatophenotypic similarities in PSGN, we carried out a Spearman's rank correlation test of gene degrees. This test showed weak, but statistically significant, positive correlations between gene degrees for the whole set of genes (p-value = 2.0E−07, r = 0.15 for HDN; p-value = 3.2E−08, r = 0.16 for ODN) when PIN was compared to PSGN. No significant correlations were found when either MGN or FSGN were compared to PSGN (Table S9). The values for the different subsets obtained in this analysis clearly show that only physical interactions bear some relation with the abundance of pathophenotypic similarities in pleiotropic genes associated with monogenic diseases (MD-PG). Accordingly, mutations in MD-PG genes seem to “diverge” disturbances more efficiently by protein-protein interactions that determine a pathophenotypic and functional relationship between genes. This result suggests that these genes co-participate in different variants of a given disease and there are functional co-dependencies among them. Thus, we proceeded to assess whether the specificity of the pathophenotypic similarity between genes depends on their type of biological interaction. For that reason, we performed a validation analysis through receiver operating characteristic (ROC) curves to prove the signal in pathophenotypic similarities produced by each biomolecular interactome in PSGN (Figure 7). PIN and MGN showed higher average areas under the ROC curves (AUC values of 0.77 and 0.76, respectively) than functional interactions with an average AUC of 0.66 (Figure 7). Both biochemical interactomes have a strong signal, as depicted by ROC far from the straight line representing randomness (Figure 7). This observation reinforces the idea that strong synergies occur between genes involved in biochemical interactions. The functional network (Figure 7) also shows a signal clearly departed from the straight line representing randomness that is consistent with previous works [27]. However, one should be aware that there is always some degree of nonspecific relationships that can introduce noise in this kind of analysis.

#### Merging modular components of MSUD using pathophenotypic similarity

**Figure 8:**
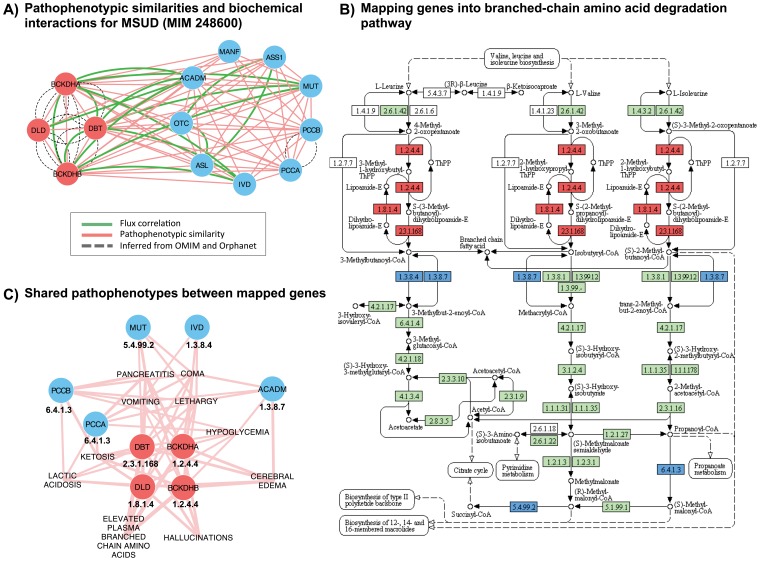
Maple syrup urine disease pathological and metabolic interactions. In red genes associated with MSUD and in blue pathophenotypic similar genes. (A) Pathophenotypic similarity gene sub-network for MSUD causing genes. It can be noteworthy that there are no inferred relationships between MSUD genes and the rest. (B) Map of branched-chain amino acid degradation pathway from. This map has been extracted from the Kyoto Encyclopedia of Genes and Genomes (KEGG, hsa:00280) developed by Kanehisa Laboratories. Enzymes encoded by human genes are in green. (C) Pathophenotypes shared between genes in the same metabolic module.

We analyzed a metabolic disorder named as maple syrup urine disease (MSUD, MIM 248600). MSUD is a genetic disease grouped into aminoacidurias and caused by a decreased activity of the branched-chain alpha-ketoacid dehydrogenase (BCKD) complex. It catalyzes the first steps for the degradation of branched-chain amino acids (valine, leucine and isoleucine). This enzymatic complex has three subunits (E1, E2, and E3) encoded by four different genes BCKDHA-E1A (Entrez GeneID 593), BCKDHB-E1B (Entrez GeneID 594), DBT-E2 (Entrez GeneID 1629), and DLD-E3 (Entrez GeneID 1738). This inborn error of metabolism is genetically and phenotypically well characterized [47]. The classical clinical features associated with MSDU are: maple syrup odor in cerumen (hours after birth), increased levels of branched -chain amino-acids (valine, leucine and isoleucine), ketonuria, signs of deepening encephalopathy, coma and central respiratory failure. We retrieved a map of all pathophenotypes annotated for MSUD-causing genes (Figure S3). From PSGN, we retrieved all gene pairs including at least one of the MSUD causing genes, but before we removed a dense cluster linked to DLD due to Leigh syndrome (Figure 8 A). Some of the resulting genes also present direct or non-direct metabolic flux correlations with BCKDHA, BCKDHB, DBT or DLD (Figure 8 A) and most of them take part in different reactions of the valine, leucine and isoleucine degradation pathway (Figure 8 B). This evidence that integrating functional co-dependencies and pathophenotypic similarities merge apparently non-related genes into a module of the molecular pathobiology. Furthermore, we can breakdown the module relationships to map shared pathophenotypes between genes (Figure 8 C). For instance, IVD and ACADM are genes included in MD-MG subsets for both HDN and ODN. However, in this sub-network (Figure 8 A) we detect that they are sharing pathophenotypes with MSUD genes (Figure 8 C). It is possible to identify the set of the most specific pathophenotypes for MSUD, elevated plasma branched chain aminoacids or hallucinations. In addition, PCCA and PCCB appear with similar clinical biochemistry parameters highly correlated with MSUD, such as high levels of lactic acid and ketone bodies (Figure 8 C). In contrast, other pathophenotypes point to disorders at a systemic or pathophysiological level, such as cerebral edema, pancreatitis, lethargy and coma (Figure 8 C). Nevertheless, these genes are grouped in the same biological context (Figure 8 B) and, it is important to remark, that all of them are in the mitochondrial matrix.

This metabolic syndrome illustrates the potentials of PSGN. This network provides novel pathological similarities between genes and outlines the pathobiology and functional context of disease-causing genes using metabolic interactions.

#### Overlapped physical and pathophenotypic interactions disregarded in unipartite projections

**Figure 9:**
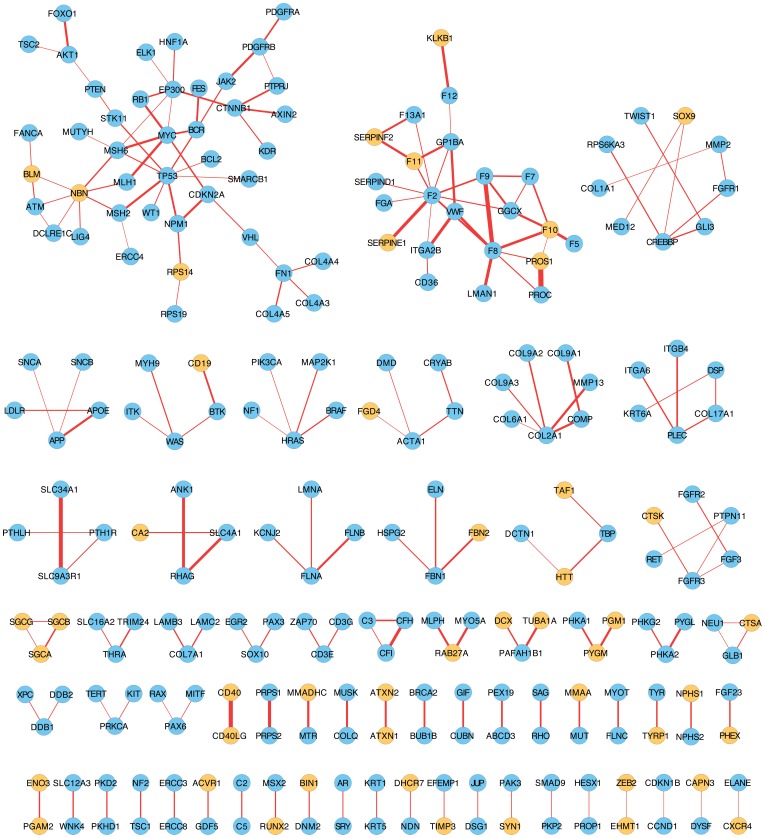
Physical interactions between genes with similar phenotypic lost in the current networks of diseases. This figure is the result of the difference of the resulting intersection between PSGN and PIN after removing those interactions present in HDGN and ODGN. Those genes that are MD-MG in HDN and ODN have been coloured in orangeThese genes indicate that they present underlying pathophenotypical relationships with other genes that had been disregarded by the inference of shared disease genes.

Finally, given the relevance of the physical interactions, we carried out a manual exploration of the intersection between PIN and PSGN. This is to remove all those gene-to-gene edges in both HDGN and ODGN from the resulting intersection. This resulted in the selection of all the disregarded relationships between genes in unipartite projections of diseasomes that are phenotypically and physically related (Figure 9 and Table S10). Therefore, tuning the balance between the “noise” and the confidence of interactions may improve the predictive power of new disease-related genes using network medicine approaches based on pathophenotypic term.

### Conclusions

Current studies in medical genetics are mainly centered in establishing associations among diseases and genetic variations for personalized medicine. Many of these genetic variations are located in intragenic regions of DNA and they constitute the basic data to build disease-causing gene networks [10], [11]. These networks are useful to find new genetic interactions between diseases, as well as to predict the influence of gene functions in existing pathologies [48]–[50]. In the present work, we have classified the different patterns of gene-disease associations in four subsets according to two different criteria (MD-MG, MD-PG, PD-MG, PD-PG, as depicted in Figure 1C). This is in contrast to previously published works in which only one criterion was used, either specific and shared genes by diseases [30] or monogenic or polygenic disease-causing genes [31], [51]. Our findings indicate that the inferred associations are insufficient to describe properly both interactions among diseases and among genes. This effect can be easily observed when analyzing bipartite graphs composed of gene-to-disease edges. In these networks, more than 30% of the genes participate in “bi-univocal” relationships (that is, genes associated exclusively with a single disease). This specificity can be useful for diagnostics, but it makes it more difficult to establish groups or to identify interactions among diseases. On the other hand, our results have also uncovered an enrichment of metabolic genes in bi-univocal subsets, as well as an enrichment of essential genes in pleiotropic subsets. The lack of cellular and molecular phenotyping platforms constrains the possibility to detect shared features among pathologies. Consequently, this reduces the possibilities of generating new knowledge on the molecular bases of the pathophenotypic profiles, to distinguish classes and subclasses of a given disease more precisely [7], [11], [26]. However, medical semantics remains the standard tool to establish the sets of observed clinical features associated with pathologies. In the case of diseases with predominantly genetic origins, pathophenotypes are usually very conserved among patients. We have shown that pathophenotypic similarity gene networks can be a great resource to uncover the molecular mechanisms involved in the responses of organisms to genetic disturbances. For instance, it shows to be useful to merge biomolecular components involved in a same pathological process like MSUD.

In the future, network integration and standardization of molecular and cellular phenotypes could improve the understanding of the evolutionary mechanisms involved in pathological processes. Further experimental and analytical efforts in this direction are warranted.

## Supporting Information

Figure S1 **Schematic representation of the workflow of essential steps followed in this study: building network processes, optimal statistical threshold selection, network comparisons, topological analysis and ROC curve construction.**
(PDF)

Figure S2 **Spearman correlation between the number of pathophenotypes per gene and the average pathophenotypic similarity per gene for PSGN genes.**
(PDF)

Figure S3 **Graph of the pathophenotypes annotated to maple syrup urine syndrome. Parental nodes are close to the root in the human phenotype ontology and, therefore, with lower specificity. In contrast, child nodes are the most informative and specific pathological phenotypes.**
(PDF)

Table S1 **Bipartite and unipartite projections of the updated version of the human diseases network.**
(XLS)

Table S2 **Bipartite and unipartite projections of the updated version of the orphan disease network.**
(XLS)

Table S3 **Different gene subsets in the human diseases network following proposed classification.**
(XLS)

Table S4 **Different gene subsets in the orphan diseases network following proposed classification.**
(XLS)

Table S5 **Different biomolecular interactomes based on physical, metabolic and functional interactions.**
(XLS)

Table S6 **Biomolecular interactome and PSGN sub-networks after nodal intersections.**
(XLS)

Table S7 **Pathophenotypic similarity gene network.**
(XLS)

Table S8 **Spearman correlations between gene degrees in PSGN and HDGN/ODGN.**
(PDF)

Table S9 **Spearman correlation between gene degrees in PSGN and biomolecular interactomes.**
(PDF)

Table S10 **Network intersection between PSGN and PIN removing inferred gene-to-gene associations.**
(XLS)

Methods S1 (PDF)
