# Correction: Global Analysis of the Human Pathophenotypic Similarity Gene Network Merges Disease Module Components

**DOI:** 10.1371/annotation/03fb5f25-21c7-4dfb-9d74-00a22f160bd6

**Published:** 2013-06-18

**Authors:** Armando Reyes-Palomares, Rocío Rodríguez-López, Juan A. G. Ranea, Francisca Sánchez-Jiménez, Miguel Angel Medina

The name of the fourth author was spelled incorrectly. The correct name is: Francisca Sánchez-Jiménez. The correct abbreviation in the Contributions section is FS-J. The correct citation is: Reyes-Palomares A, Rodríguez-López R, Ranea JAG, Sánchez-Jiménez F, Medina MA (2013) Global Analysis of the Human Pathophenotypic Similarity Gene Network Merges Disease Module Components. PLoS ONE 8(2): e56653. doi:10.1371/journal.pone.0056653. 

