# Supplementary material for: Global Analysis of the Human Pathophenotypic Similarity Gene Network Merges Disease Module Components
Source: PLoS One. 2013 Feb 21;8(2):e56653. doi: 10.1371/journal.pone.0056653 (PMC3578923; doi:10.1371/journal.pone.0056653)
Supplement: Figure S1 — Schematic representation of the workflow of essential steps followed in this study: building network processes, optimal statistical threshold selection, network comparisons, topological analysis and ROC curve construction. (PDF) [file pone.0056653.s001.pdf]

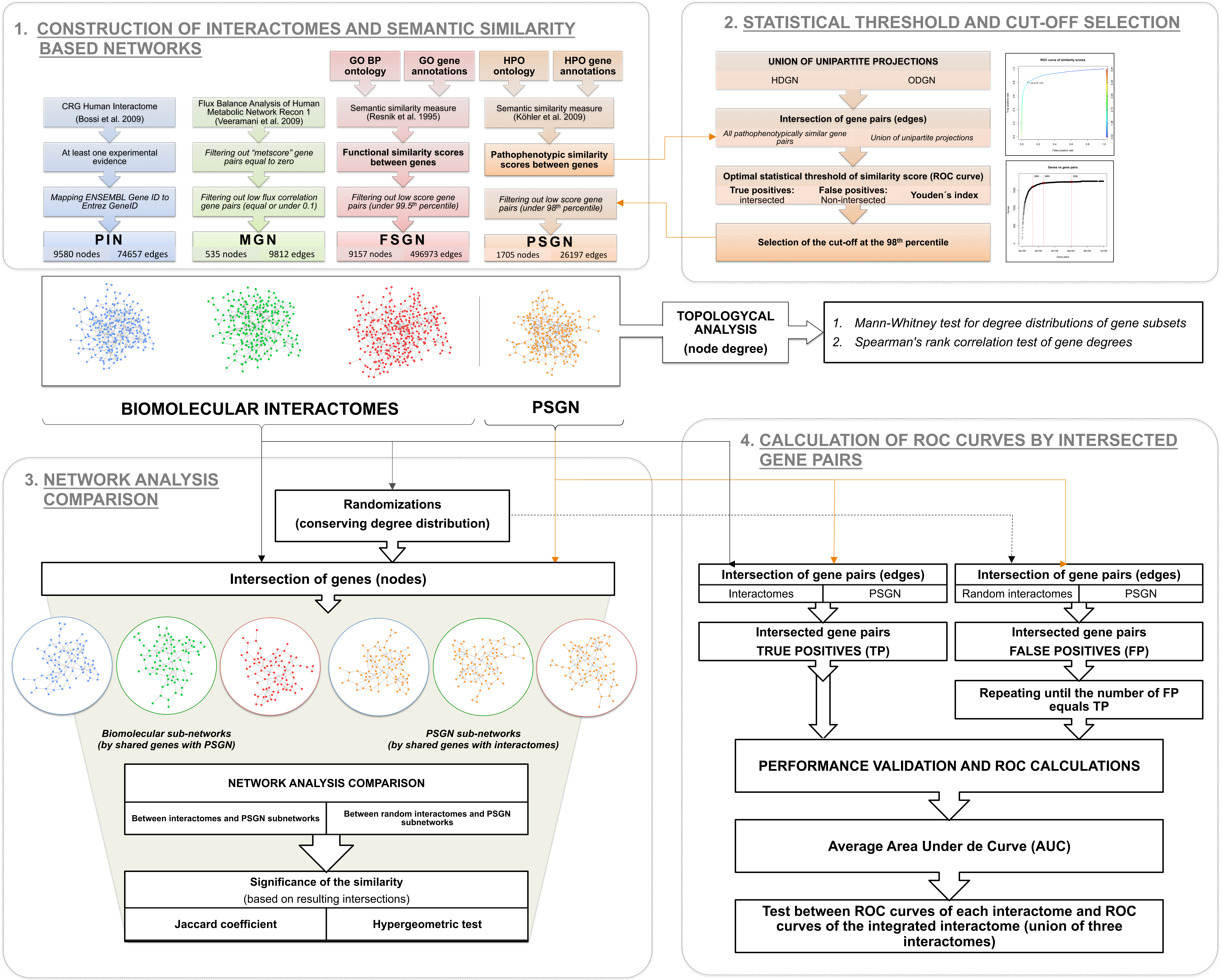
1. CONSTRUCTION OF INTERACTOMES AND SEMANTIC SIMILARITY BASED NETWORKS
GO BP ontology
GO gene annotations
HPO ontology
HPO gene annotations
CRG Human Interactome (Bossi et al. 2009)
At least one experimental evidence
Mapping ENSEMBL Gene ID to Entrez GeneID
PIN
9580 nodes 74657 edges
Flux Balance Analysis of Human Metabolic Network Recon 1 (Veeramani et al. 2009)
Filtering out "metscore" gene pairs equal to zero
Filtering out low flux correlation gene pairs (equal or under 0.1)
MGN
535 nodes 9812 edges
Semantic similarity measure (Resnik et al. 1995)
Functional similarity scores between genes
Filtering out low score gene pairs (under 99.5th percentile)
FSGN
9157 nodes 496973 edges
Semantic similarity measure (Köhler et al. 2009)
Pathophenotypic similarity scores between genes
Filtering out low score gene pairs (under 98th percentile)
PSGN
1705 nodes 26197 edges
2. STATISTICAL THRESHOLD AND CUT-OFF SELECTION
UNION OF UNIPARTITE PROJECTIONS
HDGN
ODGN
Intersection of gene pairs (edges)
All pathophenotypically similar gene pairs
Union of unipartite projections
Optimal statistical threshold of similarity score (ROC curve)
True positives: intersected
False positives: Non-intersected
Youden´s index
Selection of the cut-off at the 98th percentile
TOPOLOGYCAL ANALYSIS (node degree)
1. Mann-Whitney test for degree distributions of gene subsets
2. Spearman's rank correlation test of gene degrees
BIOMOLECULAR INTERACTOMES
PSGN
3. NETWORK ANALYSIS COMPARISON
Randomizations (conserving degree distribution)
Intersection of genes (nodes)
Biomolecular sub-networks (by shared genes with PSGN)
PSGN sub-networks (by shared genes with interactomes)
NETWORK ANALYSIS COMPARISON
Between interactomes and PSGN subnetworks
Between random interactomes and PSGN subnetworks
Significance of the similarity (based on resulting intersections)
Jaccard coefficient
Hypergeometric test
4. CALCULATION OF ROC CURVES BY INTERSECTED GENE PAIRS
Intersection of gene pairs (edges)
Interactomes
PSGN
Intersected gene pairs TRUE POSITIVES (TP)
Intersection of gene pairs (edges)
Random interactomes
PSGN
Intersected gene pairs FALSE POSITIVES (FP)
Repeating until the number of FP equals TP
PERFORMANCE VALIDATION AND ROC CALCULATIONS
Average Area Under de Curve (AUC)
Test between ROC curves of each interactome and ROC curves of the integrated interactome (union of three interactomes)
