# Supplementary figures and images for: Global Analysis of the Human Pathophenotypic Similarity Gene Network Merges Disease Module Components
Source: PLoS One. 2013 Feb 21;8(2):e56653. doi: 10.1371/journal.pone.0056653 (PMC3578923; doi:10.1371/journal.pone.0056653)

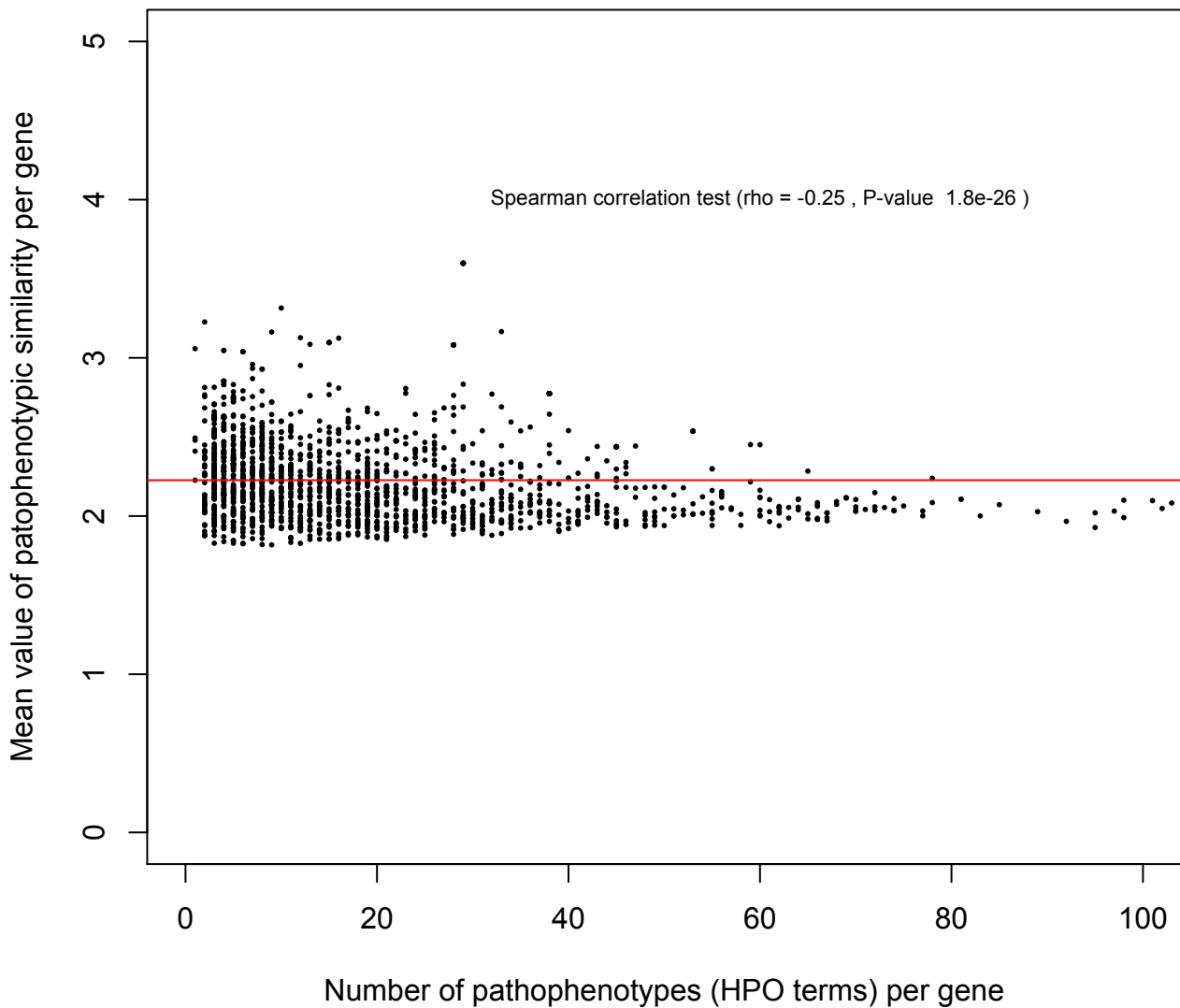

Supplement: Figure S2 — Spearman correlation between the number of pathophenotypes per gene and the average pathophenotypic similarity per gene for PSGN genes. (PDF) [file pone.0056653.s002.pdf]

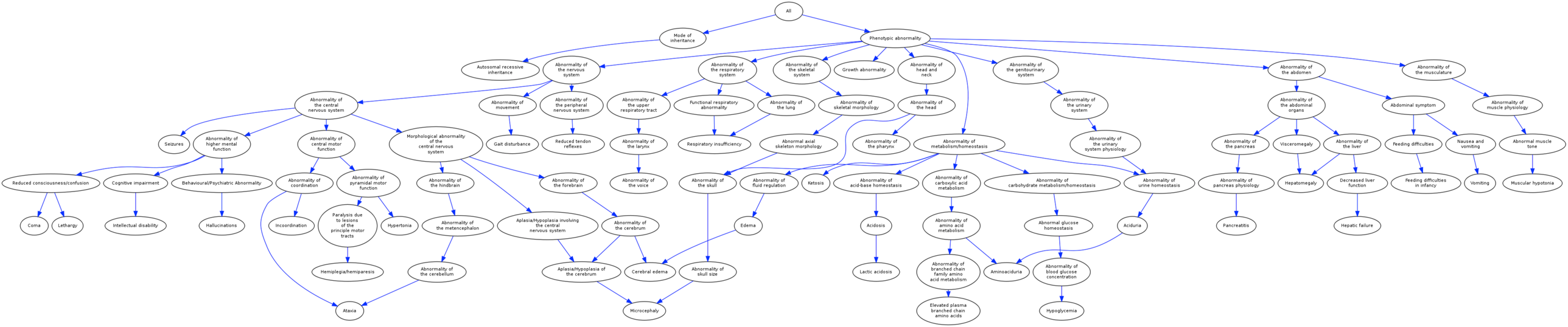

Supplement: Figure S3 — Graph of the pathophenotypes annotated to maple syrup urine syndrome. Parental nodes are close to the root in the human phenotype ontology and, therefore, with lower specificity. In contrast, child nodes are the most informative and specific pathological phenotypes. (PDF) [file pone.0056653.s003.pdf]
