# Supplementary material for: Global Analysis of the Human Pathophenotypic Similarity Gene Network Merges Disease Module Components
Source: PLoS One. 2013 Feb 21;8(2):e56653. doi: 10.1371/journal.pone.0056653 (PMC3578923; doi:10.1371/journal.pone.0056653)
Supplement: Table S8 — Spearman correlations between gene degrees in PSGN and HDGN/ODGN. (PDF) [file pone.0056653.s011.pdf]

Table S8. Spearman correlations between gene degrees in PSGN and HDGN/ODGN

	HDGN			ODGN		
**Class**	**n (genes)**	r_s	**P-value**	**n (genes)**	r_s	**P-value**
MD-MG[a]	-	-	-	-	-	-
MD-PG	247	0.06	3.4E-01	280	0.11	6.6E-02
PD-MG	226	0.26	7.3E-05	446	0.25	7.4E-08
PD-PG[b]	303	0.06	3.2E-01	485	0.13	4.2E-03
All genes[c]	528	0.17	9.2E-05	931	0.22	1.0E-11

[a] Biunivocal genes are not present in diseases causing gene networks.
[b] Pleiotropic genes associated with at least one polygenic diseases.
[c] All intersected genes between PSGN and HDGN or ODGN respectively.
