# Supplementary material for: Global Analysis of the Human Pathophenotypic Similarity Gene Network Merges Disease Module Components
Source: PLoS One. 2013 Feb 21;8(2):e56653. doi: 10.1371/journal.pone.0056653 (PMC3578923; doi:10.1371/journal.pone.0056653)
Supplement: Table S9 — Spearman correlation between gene degrees in PSGN and biomolecular interactomes. (PDF) [file pone.0056653.s012.pdf]

Table S9. Spearman correlation between gene degrees in PSGN and biomolecular interactomes

		PIN			MGN			FGN		
**Network**	**Class**	**n (genes)**	r_s	**P-value**	**n (genes)**	r_s	**P-value**	**n (genes)**	r_s	**P-value**
HDN	MD-MG	555	0.13	2.9E-03	87	-0.01	9.4E-01	634	0.01	7.7E-01
HDN	MD-PG	420	0.18	2.6E-04	31	-0.02	9.3E-01	456	-0.04	4.0E-01
HDN	PD-MG	158	0.06	4.5E-01	29	0.37	5.0E-02	175	-0.06	4.3E-01
HDN	PD-PG[b]	247	0.10	1.0E-01	14	-0.25	3.9E-01	267	-0.12	6.0E-02
HDN	All genes in HDN[c]	1175	0.15	2.0E-07	150	0.10	2.4E-01	1313	0.01	6.5E-01
ODN	MD-MG	364	0.11	3.0E-02	68	0.02	8.7E-01	411	0.01	9.3E-01
ODN	MD-PG	312	0.21	2.2E-04	26	-0.23	2.7E-01	340	-0.10	6.3E-02
ODN	PD-MG	322	0.18	1.3E-03	40	0.16	3.4E-01	354	-0.04	4.3E-01
ODN	PD-PG[b]	384	0.13	1.4E-02	28	0.01	9.5E-01	423	-0.01	9.3E-01
ODN	All genes in ODN[c]	1151	0.16	3.2E-08	149	0.07	3.9E-01	1279	0.01	5.9E-01

[a] Network based on functional similarities from biological processes branch of Gene Ontology.
[b] Pleiotropic genes associated with at least one polygenic diseases.
[c] All intersected genes between the respective biomolecular interactome and PSGN.
