# Supplementary material for: Global Analysis of the Human Pathophenotypic Similarity Gene Network Merges Disease Module Components
Source: PLoS One. 2013 Feb 21;8(2):e56653. doi: 10.1371/journal.pone.0056653 (PMC3578923; doi:10.1371/journal.pone.0056653)
Supplement: Methods S1 — (PDF) [file pone.0056653.s014.pdf]

Supplementary methods and discussion section for

“Global Analysis of the Human Pathophenotypic Similarity Gene Network Merges Disease Module Components”

Armando Reyes-Palomares[1,2], Rocio Rodríguez-López[1,2], Juan AG Ranea[1,2], Francisca Sánchez Jiménez[1,2], Miguel Angel Medina[1,2]

[1]Department of Molecular Biology and Biochemistry, Faculty of Sciences, University of Málaga, E-29071 Málaga, Spain

[2]CIBER de Enfermedades Raras (CIBERER), E-29071 Málaga, Spain

[*]To whom correspondence should be addressed.

Email: medina@uma.es

Procedures to select the similarity score cutoff

1. Introduction

A systematic methodology to identify significant semantic similarities (similarity scores) has not been yet established. We therefore propose a few systematic steps to set aside what can be considered as significant similarity scores, in agreement to the current genetic association and ontological structure knowledge. The semantic similarity proposed by Resnik computes as informative is each term [1]. The information content (also known by IC) of an ontological term depends on its relative frequency, the number of annotations of a term respect to the whole set of annotations (corpus). It means that a high of informativeness indicates more specificity and fewer annotations. Hence, the similarity between terms will be assessed calculating the IC of the most informative common ancestor (MICA) derived from the ontological structure.

We have calculated all similarity scores (pathophenotypic similarities) between annotated genes in the Human Phenotype Ontology (HPO) [2]. For this, we used an IC-based measure [3] resulting in 1309578 similarity scores between 1812 genes, excluding zero scores. In this study, genes are annotated with a set of HPO terms describing the pathological processes related to them by genetic association studies. Accordingly, the computed scores are themselves probabilistic estimations of the pathophenotypic similarity between genes respect to the total number of pathological phenotypes (HPO terms) comprehended in the study.

In this case, the percentile can be considered as a suitable parameter to decide a level of significance for all ranked scores. However, it is assumed that most of the computed pathophenotypic relationships between genes are non-informative although they are statistically recognizable by their similarity score. Furthermore, HPO is a controlled vocabulary that has been manually revised and structured as a direct acyclic graph (DAG) [4]. Consequently, the similarity scores depends on the current knowledge represented in HPO, where some relationships can be missing and general domains cluster many terms. It means that noise could be present in any computed similarity score using biomedical ontologies [5], so the identification of an optimal statistical threshold remains as the greatest difficulty. This is to set the limit of similarity score

from which gene-gene relationships are significantly informative. Therefore, we have two main purposes to identify this optimal statistical threshold and, once it has been assessed, to select the most appropriate cutoff of similarity score.

2. Procedure

We started by analyzing the properties of all the computed pathophenotypic similarities. The similarity score distribution reveals that lower values are clearly more frequent (more probable) than higher ones (Figure 1). Indeed, it is in line with the information content computed by the semantic similarity measurement applied for this work [6], which also uses the relative frequency.

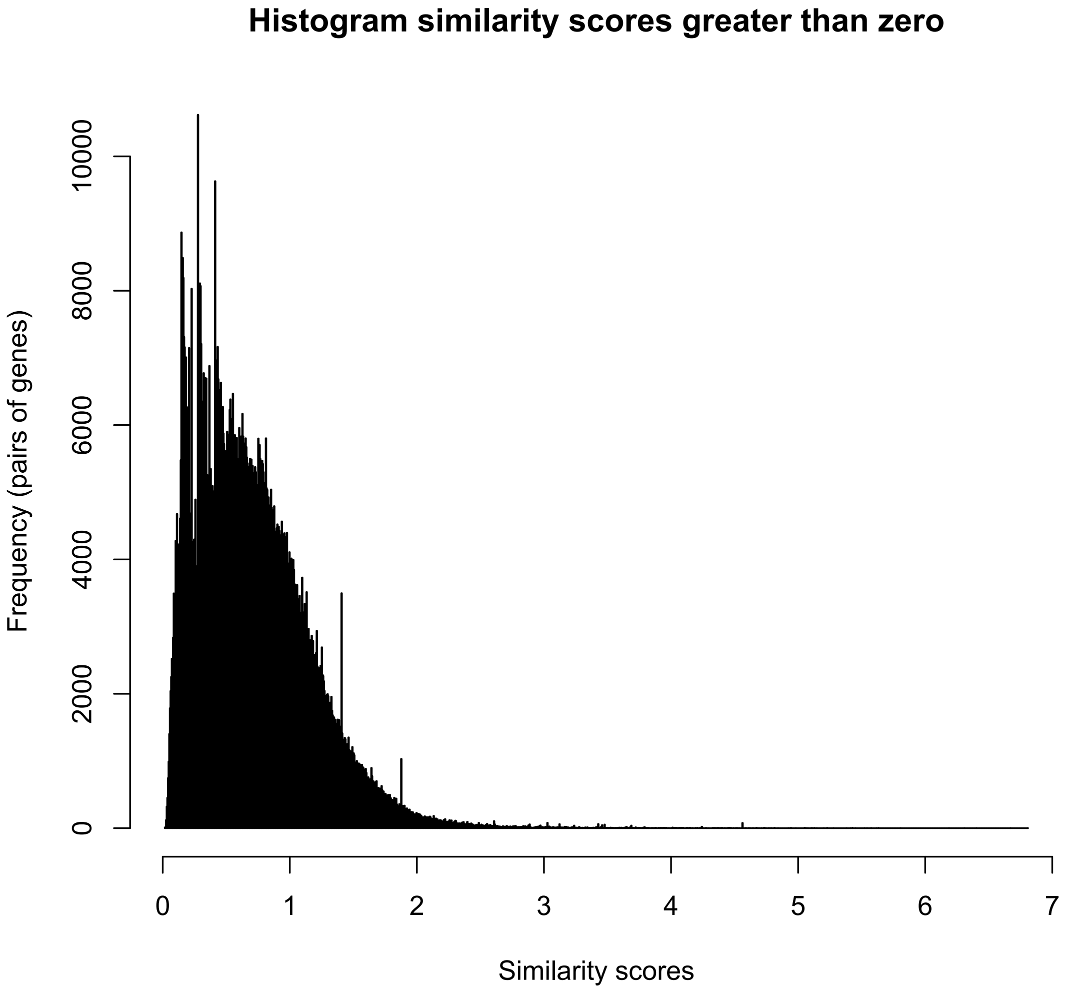

Figure 1. Histogram of gene pairs per similarity score. The number of gene pairs for each similarity score after to remove zero scores. It can be note that a high frequency for bars located the region of lower similarity scores.

Figure 1 represents the number of gene pairs counted for each score. In some cases, similarity scores reach more than 10000 pairs of genes and they are located along the region of smaller values. This may be due to the fact that low similarity scores have been computed by HPO terms close to the root of the ontology conforming general domains (clusters) of non-specific pathophenotypic similarities between genes. Therefore, these domains lack of significant information to become constituent elements of the pathophenotypic gene similarity network (PSGN). These clustering effects can be observed by analyzing the number of resulting genes at different cutoffs among the whole range of similarity scores. Thus we used 1000 different cutoffs to study in details variations on this distribution. For instance, it could be appreciated that the number of participating genes decreases as the similarity score cut-off increases (Figure 2); what indicates that genes are not taken into account if they are not participating in any relationship over the threshold.

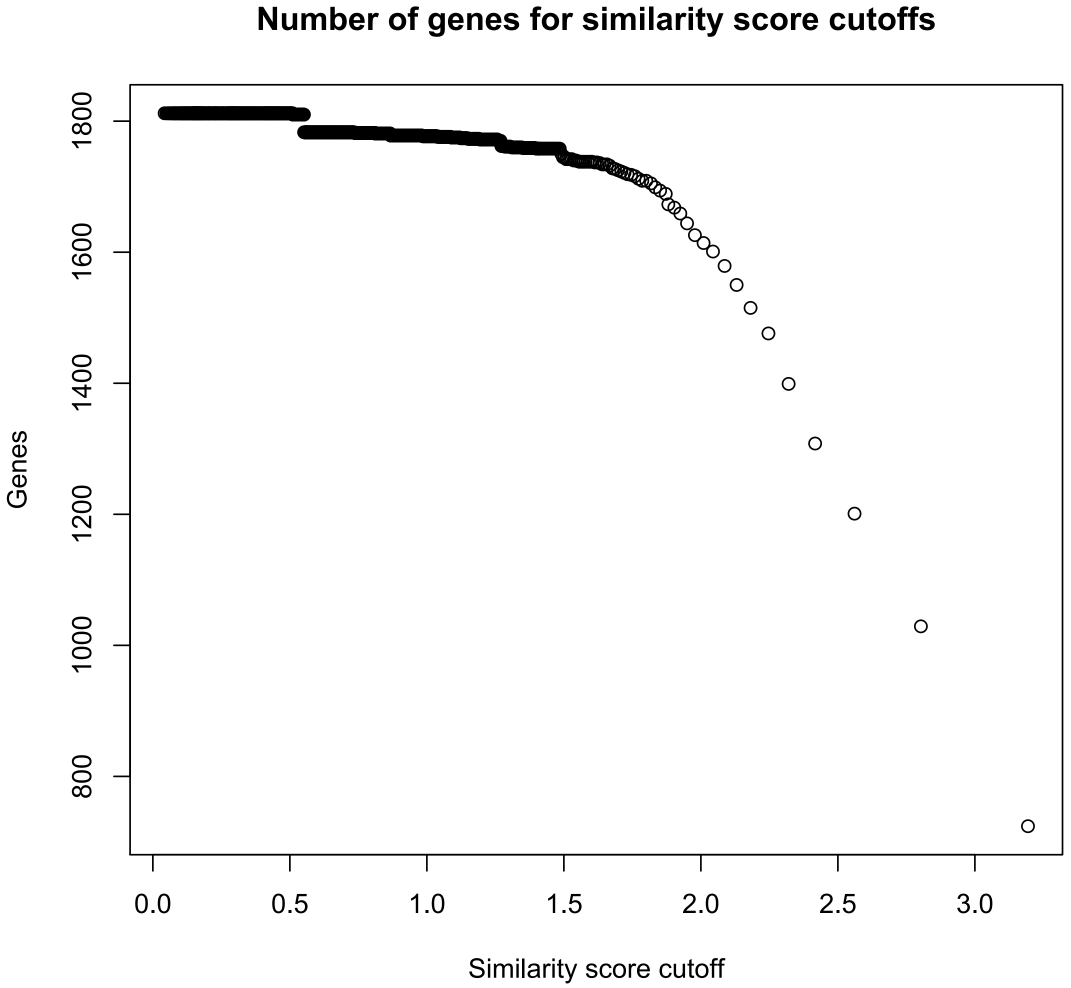

Figure 2. Number of genes counted at each similarity score cutoffs. One thousand equidistant cutoffs were established along the whole range of similarity scores. Subsequently, gene pairs with a score equal or greater than the used cutoff were selected and resulting genes were counted.

As can be noted, the clustering effects are more evident for higher similarity scores where little variations lead to introduce strong differences in the number of genes. In contrast, the number of genes is well conserved when using low similarity scores as cutoffs. However, along this flat area, we observe abrupt shifts in the number of genes, whose can be due to discard scores associated with HPO terms grouping many genes (group of genes sharing HPO annotations). The number of genes is stable but the gene pairs decreased exponentially until it reaches scores higher than approximately 1.5 (Figure 3).

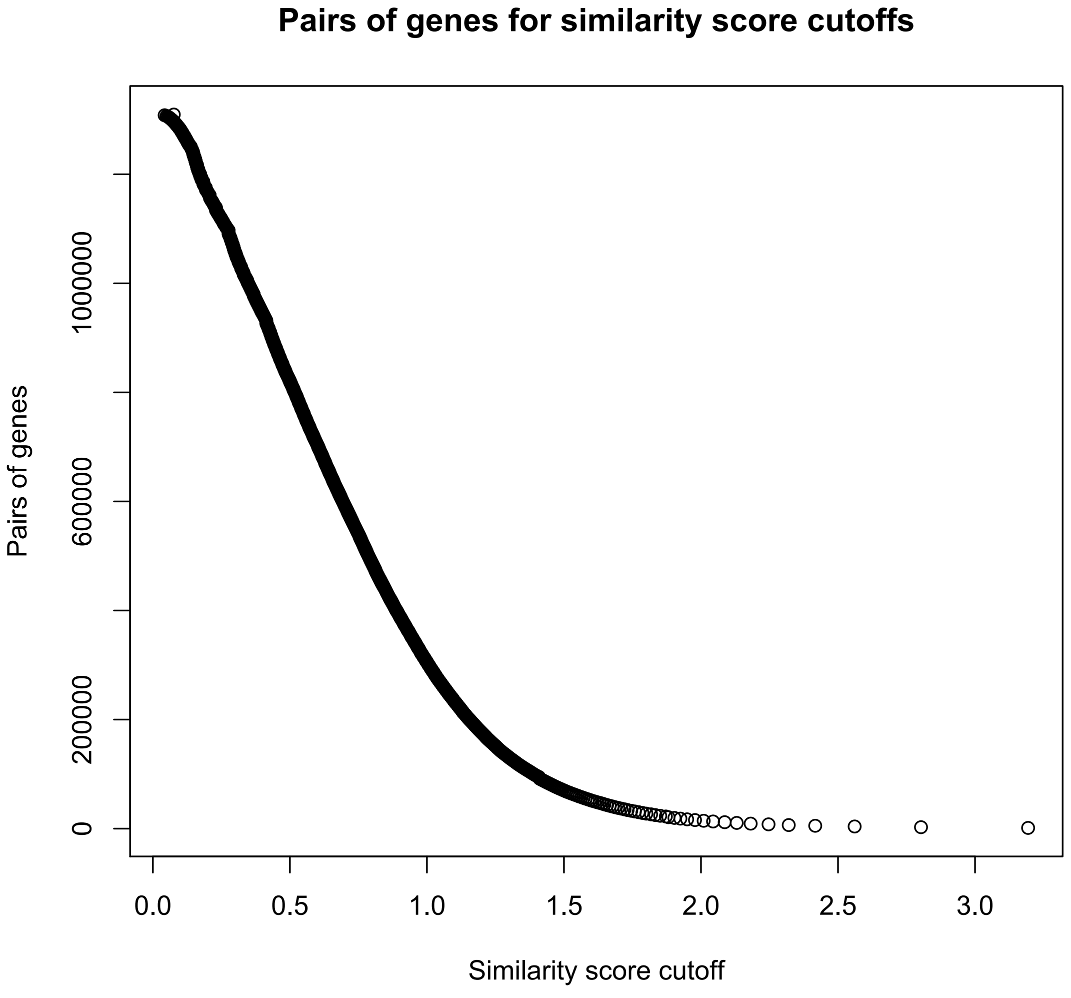

Figure 3. Number of gene pairs for similarity scores cutoff. One thousand equidistant cutoffs were established along the whole range of similarity scores. All gene pairs with a score equal or greater than the used cutoff were selected. Three types of areas can be approximately defined: vertical asymptote (approx. from 0 to 1.0 cutoffs), turning point of the curvature (approx. from 1.0 to 2.0 cutoffs) and horizontal asymptote (approx. from 2.0 to 1.0 cutoffs).

Figure 3 represents a curve with three different zones: a vertical asymptote, a curvature and a horizontal asymptote. The vertical asymptote represents a clear lineal dependency between the used cutoff and the number of gene pairs. Then, many gene-gene relationships are affected by minor variations in the similarity score during the analysis. This result can be partially explained by hierarchical dependencies between HPO terms in the ontology and suggests the exponential growth of nonspecific similarities as the score drops. On the other hand, the horizontal asymptote seems to indicate that the number of gene pairs is conserved when the cutoff has reached a certain score. But what really happens is that the specificity increases considerably with higher values of semantic similarity, what means that genes are sharply clustered. Hence, the curvature represents the threshold to distinguish from low to high signal-to-noise ratio, in the vertical and horizontal asymptote, respectively (Figure 3).

3. Results for threshold and cutoff selection

Different approaches were used to assess the biological relevance of this threshold in order to maximize noise-reduction and specificity. Subsequently, this optimal threshold should be the reference value from which to set the most appropriated cut-off to build the PSGN.

First off all, we performance a binary classifier system and illustrate it in a receiver operating characteristic (ROC) curve. This binary system was built from gene pairs showing pathophenotypic similarity (1309578 gene pairs) that were present or absent in the union of both unipartite projections (HDGN and ODGN). We joined all inferred interactions from HDGN and ODGN considering as unique the redundant ones. It should be pointed, that both unipartite projections (HDGN or ODGN) are based on genes that are associated with at least one same disease in OMIM and Orphanet, respectively. The resulting network, in turn, was compared with the dataset of pairs of genes obtained after to compute similarity scores between genes. It resulted in a cross-tabulation of overlapped and non-overlapped gene pairs to be considered respectively as true positives and false positives (Table 1).

Table 1. Binary classification based on the intersection of gene pairs

	Genes	Gene pairs
**Pathophenotypic similarities**	1812	1309578
**Unipartite projections (HDGN + ODGN)**	1760	8372
**Overlapped or True Positives (TP)**	1064	3271
**Non-overlapped or False Positives (FP)**	748	1306307

This classification model has been used to predict the optimal threshold of pathophenotypic similarity score involving a pair of genes on the same pathological process (Figure 4).

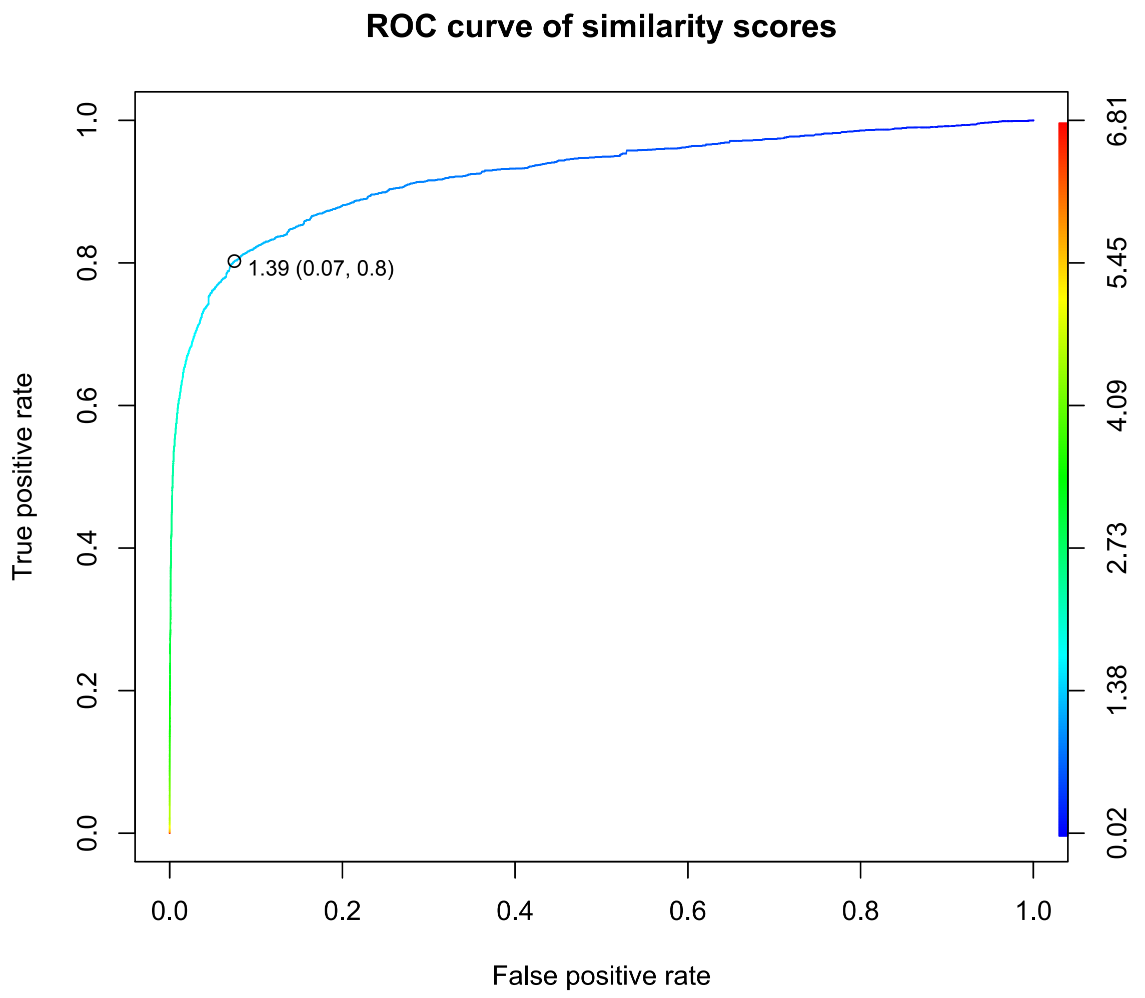

Figure 4. ROC curve and optimal threshold of pathophenotypic similarity. In this performance analysis, those pairs of genes showing pathophenotpic similarity and co-associated with at least one diseases are considered as true positives and the rest of gene pairs showing pathophenotypic similarity are considered as false positive. Black circle indicates the coordinates (0.07 and 0.8 for false and true positive rates, respectively) for the Youden's index (1.39) that determines the optimal threshold. The color palette maps for each threshold the corresponding similarity scores. ROCR [7] and pROC [8] packages were used to represent and calculate optimal threshold.

As can be appreciated from the ROC curve the pathophenotypic similarity (similarity score) is a good indicator to assess the underlying relationships of genes in a particular pathological process. The optimal threshold of similarity score that maximizes the trade-off between the rates of true and false positives is 1.39 (which corresponds to the 92th percentile, Table 2) for the Youden's index [9,10] (Figure 4). We also calculated the inflection point at the curvature in Figure 3, which can be worth as an alternative approach to locate the turning point. We fit the curve of computed similarity scores to an exponential function and the inflection point results in 1.55 corresponding to the 95th percentile (Table 2). This curve-fitting analysis was carried out in MATLAB.

Table 2. Results using top score percentiles as cutoffs

Percentile	Score	Genes	Gene pairs
**92th**	1.39[a]	1759	99777
**95th**	1.55[b]	1742	60742
**98th**	**1.82**	**1705**	**26197**
**99th**	2.04	1601	13098

[a] Patho-phenotypically meaningful threshold by ROC curve analysis.

[b] Turning point fitting similarity scores to an exponential function.

Therefore, we have two different thresholds to estimate the appropriate cutoff to be used to build the pathophenotypic similarity gene network. However, the 92th and 95th percentiles are located in regions under the influence of large clusters of non-specific similarities. For instance, we observe that similarities with scores below 1.5 show abrupt changes in the number of genes (as can be seen in Figure 2). Thus, we carried out a more detailed analysis for these regions by filtering all similarity scores below 1.39, which could be considered as the patho-phenotypically meaningful threshold (Figure 4).

In particular, the number of genes begins to drop around the value of 20.000 computed similarities scores (Figure 5). In this case, the score in the 98th percentile (1.8179) represents a reference value from which to ensure high specificity for pathophenotypic similarities without losing information. Therefore, 1.8179 corresponds to the lowest similarity value at the top 2% of all ranked scores and the most appropriate cutoff to build PSGN, for the reasons discussed above.

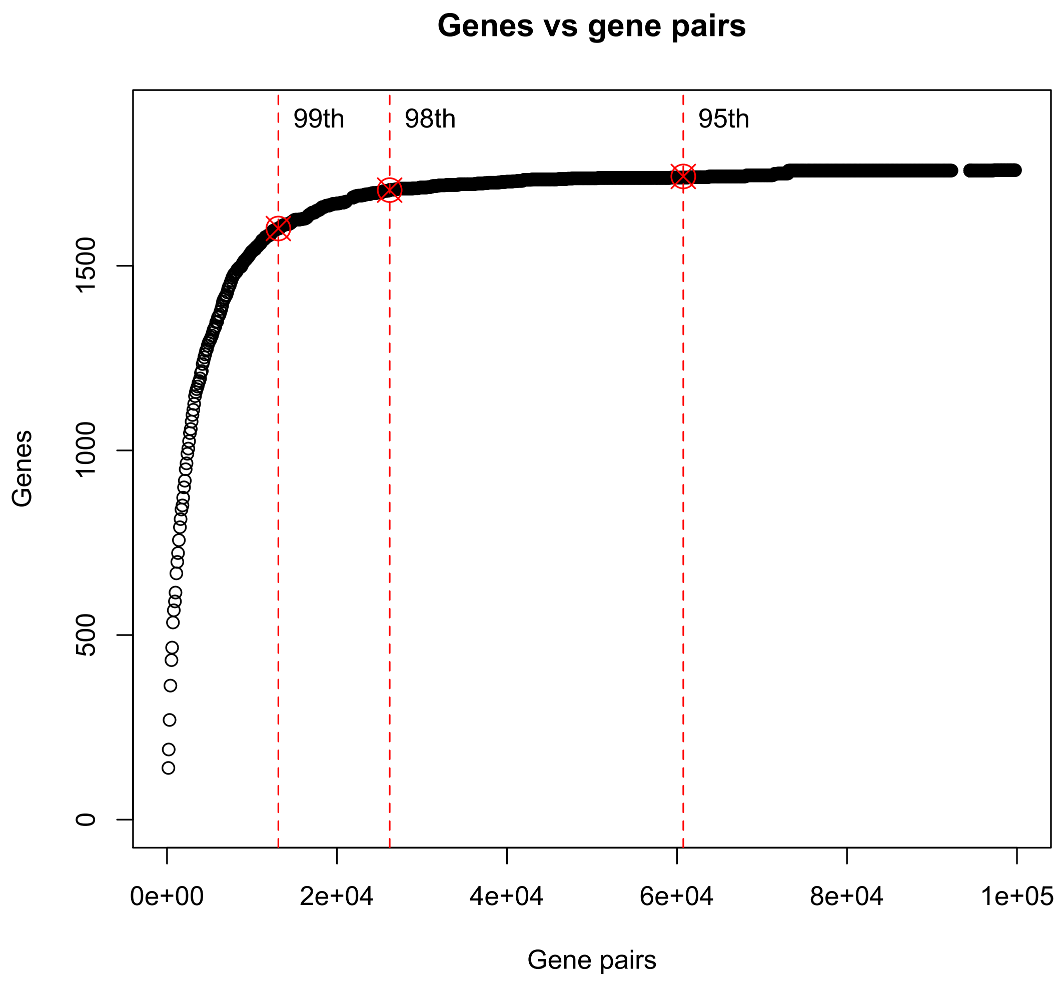

Figure 5. The number of genes and gene pairs for each similarity score cutoffs. One thousand equidistant cutoffs were established along the range of similarity scores from the 92th percentile, it means removing scores below 1.39. All gene pairs with a score equal or greater than the cutoff were selected and resulting genes were counted. Red circles and vertical lines indicate exact location for 95th, 98th and 99th percentiles.

4. References

1. Resnik P (1995) Using Information Content to Evaluate Semantic Similarity in a Taxonomy. IJCAI. pp. 448–453.
2. Robinson PN, Mundlos S (2010) The Human Phenotype Ontology. Clin Genet 77: 525–534.
3. Köhler S, Doelken SC, Rath A, Aymé S, Robinson PN (2012) Ontological phenotype standards for neurogenetics. Hum Mutat 33:1333-1339
4. Robinson PN (2012) Deep phenotyping for precision medicine. Hum Mutat 33: 777–780.
5. Pesquita C, Faria D, Falcão AO, Lord P, Couto FM (2009) Semantic Similarity in Biomedical Ontologies. PLoS Comput Biol 5: 12.

6. Köhler S, Schulz MH, Krawitz P, Bauer S, Dölken S, et al. (2009) Clinical Diagnostics in Human Genetics with Semantic Similarity Searches in Ontologies. Am J Hum Genet 85: 457–464.

7. Sing T, Sander O, Beerenwinkel N, Lengauer T (2005) ROCR: visualizing classifier performance in R. Bioinformatics 21: 3940–3941.

8. Robin X, Turck N, Hainard A, Tiberti N, Lisacek F, et al. (2011) pROC: an open-source package for R and S+ to analyze and compare ROC curves. BMC Bioinformatics 12: 77.

9. Youden WJ (1950) Index for rating diagnostic tests. Cancer 3: 32–35.

10. Perkins NJ, Schisterman EF (2006) The inconsistency of "optimal" cutpoints obtained using two criteria based on the receiver operating characteristic curve. Am J Epidemiol 163: 670–675.
